# Azide-Functionalized Naphthoxyloside as a Tool for Glycosaminoglycan Investigations

**DOI:** 10.1021/acs.bioconjchem.1c00473

**Published:** 2021-11-16

**Authors:** Daniel Willén, Roberto Mastio, Zackarias Söderlund, Sophie Manner, Gunilla Westergren-Thorsson, Emil Tykesson, Ulf Ellervik

**Affiliations:** † Centre for Analysis and Synthesis, Centre for Chemistry and Chemical Engineering, Lund University, P.O. Box 124, SE-221 00 Lund, Sweden; ‡ Department of Experimental Medical Science, Lund University, P.O. Box 117, SE-221 00 Lund, Sweden

## Abstract

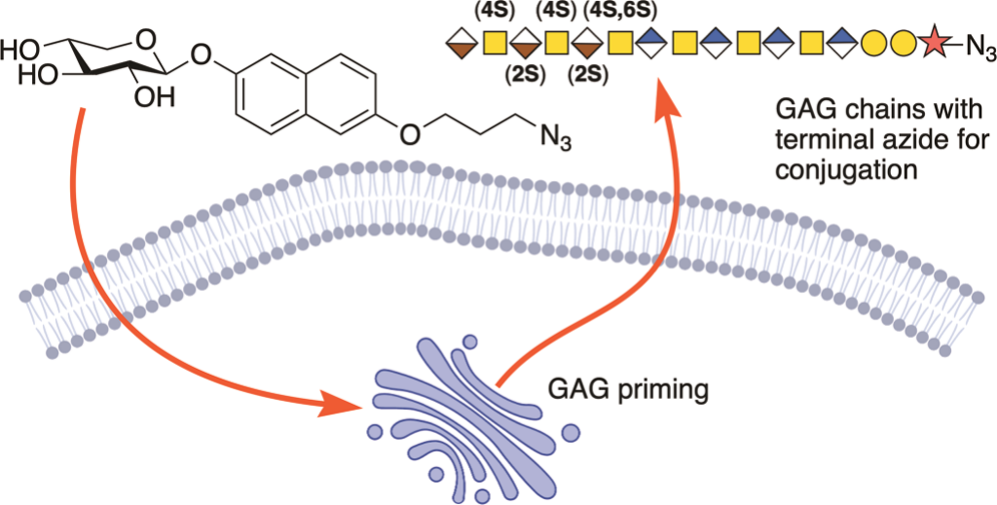

We present a xylosylated naphthoxyloside carrying a terminal azide functionality that can be used for conjugation using click chemistry. We show that this naphthoxyloside serves as a substrate for β4GalT7 and induces the formation of soluble glycosaminoglycan (GAG) chains with physiologically relevant lengths and sulfation patterns. Finally, we demonstrate its usefulness by conjugation to the Alexa Fluor 647 and TAMRA fluorophores and coupling to a surface plasmon resonance chip for interaction studies with the hepatocyte growth factor known to interact with the GAG heparan sulfate.

## Introduction

Cellular communication is essential for tissue development, growth, adhesion, coagulation, and pathophysiological processes, for example, tumor development and infection.^1^ A vital part of cell-to-cell communication is mediated by carbohydrates anchored to the cellular membranes. Proteoglycan (PG), which consists of long linear chains of alternating disaccharides, i.e., glycosaminoglycans (GAGs), is one important class of cell surface carbohydrates. The pattern of alternating disaccharides further classifies the GAGs as heparan sulfate (HS, GlcNAc(β1–4)GlcA(β1–4)) or chondroitin/dermatan sulfate (CS/DS, GalNAc(β1–4)GlcA(β1–3)). These polymers are postsynthetically sulfated and epimerized to generate a vast structural diversity.

**Figure 1:**
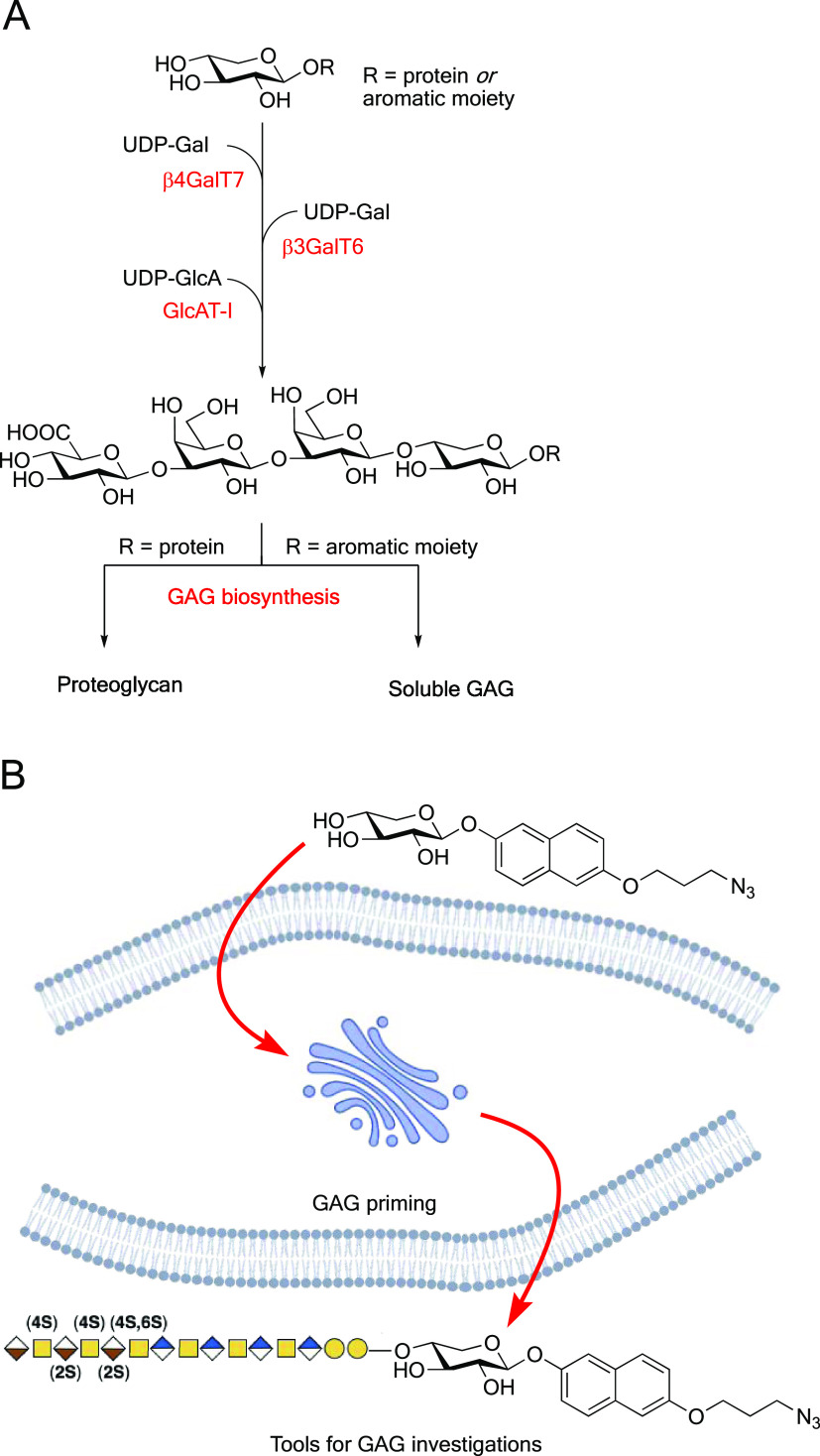
(a) Biosynthesis of the linker tetrasaccharide of HS and CS/DS from a xylosylated protein or an exogenously supplied xyloside with an aromatic aglycon. (b) Concept of this study: azide-functionalized xylosides that can prime GAGs and serve as tools for investigations of the biosynthesis and functions of GAGs.

The biosynthesis of HS and CS/DS is initiated by xylosylation of a serine residue on the parent protein followed by galactosylation by β-1,4-galactosyltransferase 7 (β4GalT7). This disaccharide is then elongated by the addition of another galactose unit and a glucuronic acid moiety to form a common linker region, i.e., GlcA(β1–3)Gal(β1–3)Gal(β1–4)Xyl β (Figure 1A).

Interestingly, the biosynthesis can be initiated by synthetic xylosides, i.e., small molecules composed of a xylose coupled to an aglycone. GAG chains primed by such xylosides are soluble and thus usually secreted into the extracellular matrix and compete with the endogenous production of PGs.

The composition of GAG chains primed by exogenously supplied xylosides is cell-specific but also, to a lesser extent, predetermined by the type of xyloside as well as the aglycon.^2^ The structure–activity relationships between aglycon and GAG structure are still not completely understood.^3^ The soluble GAG chains have been shown to have interesting properties such as antitumor effects,^4−6^ anticoagulant effects,^7,8^ lung development and regeneration,^9^ and promotion of neuronal growth.^10^

Since xylose is a relatively uncommon carbohydrate in mammalian cellular systems,^11^ apart from the GAG biosynthesis, it is only found in the Notch receptor and dystroglycan,^12^ the addition of xylosides can be used to investigate the GAG biosynthesis without disturbing other carbohydrate-mediated cellular systems. Xylosides can thus provide relatively large amounts of GAG chains that mirror the normal cell-specific PGs. These GAG chains can be used to explore the interaction with normal and tumor cells and pathogens such as bacteria and viruses.

To study the biological effects of soluble GAG chains, it is fundamental to design xylosides with aglycones that are optimized for the priming of GAG chains while also provided with a handle that can be used to make conjugates such as fluorescent probes, prodrugs, and linkers for binding to, e.g., surface plasmon resonance (SPR) sensor chips.

Recently, we presented a naphthoxyloside containing an amine function that was used in connection with a library of genetically modified knock-in/out cell lines with modifications in the GAG biosynthesis, i.e., a GAGome.^13^ The amine-functionalized naphthoxyloside induced the biosynthesis of soluble GAGs that could be used to assemble GAG microarrays. However, the amine functionality is far from optimal due to its high reactivity in cellular settings.

The Cu(I)-catalyzed 1,3-dipolar cycloaddition of azides and alkynes is one of the most famous examples of a click reaction,^14,15^ i.e., reactions aiming at the facile and rapid assembly of more complex molecules.^16^ In the early 2000s, Bertozzi and co-workers developed a copper-free version of this reaction that relies on strain promotion using a cyclooctyne, which then cleanly reacts with the azide in a reaction orthogonal to normal cellular processes.^17^ Further optimizations of the cyclooctyne gave rise to high-yielding and highly reactive compounds, i.e., DBCO, for bioconjugations.^18^

In this study, we envisioned expanding the concept of GAG primers to include a terminal azide function. We hypothesize that naphthoxylosides carrying a terminal azide function will induce the production of soluble GAG chains that reflect the cells’ natural PGs and can be conjugated with any alkyne-containing functionality and thus be used as tools to determine important roles of GAGs in various biological systems (Figure 1B).

## Results and Discussion

### Synthesis of Azide-Functionalized Naphthoxylosides

**Scheme 1:**
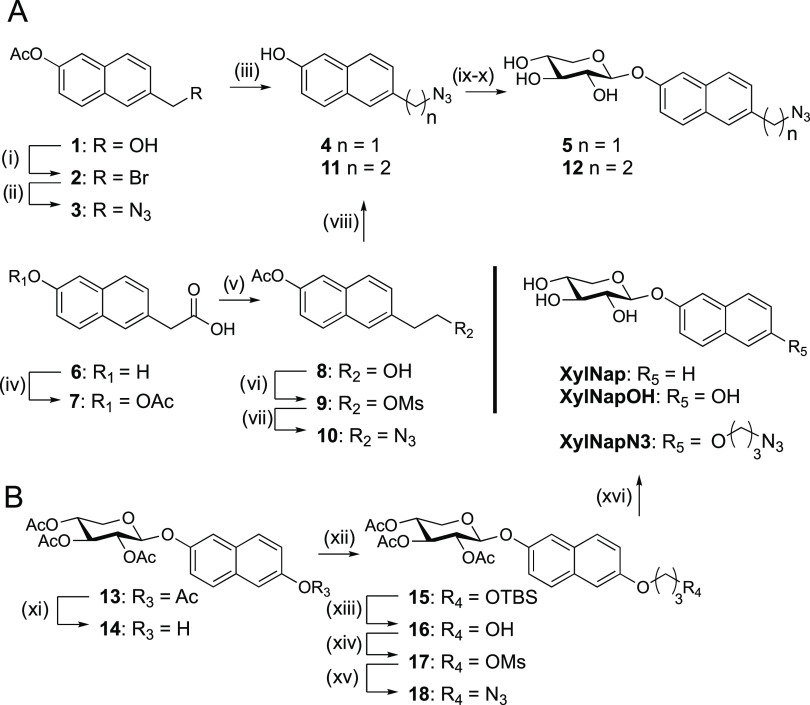
Synthesis of Target Xylosides Reagents and conditions: (i) PBr_3_, CH_2_Cl_2_, 40 °C, 1.5 h, 56%; (ii) NaN_3_, DMSO, 40 °C, 2 h, 89%; (iii) 1 M NaOMe, MeOH, r.t., 2 h, 73%; (iv) acetic anhydride, pyridine, r.t., 24 h, 68%; (v) 2 M BH_3_·THF, THF, 0 °C to r.t., 20 h, 68%; (vi) MsCl, pyridine, 0 °C, 3 h, 79%; (vii) NaN_3_, DMF, 0 °C to r.t., 30 h, 74%; (viii) 1 M NaOMe, MeOH, r.t., 2 h, 89%; (ix) peracetylated xylose, BF_3_·OEt_2_, Et_3_N, CH_2_Cl_2_, 0 °C to r.t., then (x) 1 M NaOMe, MeOH, r.t., 1 h, **5**: 13% over two steps; **12**: 16% over two steps; (xi) NH_4_OAc, THF, MeOH, H_2_O, o.n., 40 °C, 84%; (xii) 3-(*tert*-butyldimethylsilyloxy)propyl bromide. K_2_CO_3_, DMF, Ar(g), o.n., 40 °C; then (xiii) HCl, MeOH, 30 min, r.t., 63% over two steps; (xiv) MsCl, pyridine, 1,5 h, 0 °C to r.t., 89%; (xv) NaN_3_, DMF, 30 min, MW heating at 90 °C, 88%; and (xvi) K_2_CO_3_, MeOH, 1.5 h, r.t., 84%.

As a first attempt toward azide-functionalized naphthoxylosides, we envisioned compounds **5** and **12**, where the azide function is connected to the naphthol moiety via a short aliphatic linker. Starting from previously published materials **1**(19) and commercially available **6**, the azides were introduced using nucleophilic displacement, furbishing **10** and **2**, respectively (Scheme 1A). Deacetylation then gave **4** and **11**, which were xylosylated and deprotected to give **5** and **12**, respectively. These reactions were surprisingly low-yielding, and we experienced purification problems. Furthermore, **5** and **12** were unstable in the buffer used for biological investigations. It is known that azides, in the presence of Brønstedt and Lewis acids, can give rise to rearrangements and cyclization reactions.^20^ These envisioned side reactions between the aromatic ring and the azide functionality rendered compounds **5** and **12** less suitable for further evaluation.

We decided to increase the linker length to avoid these side reactions, allowing synthetically simpler constructs, e.g., ethers. Conceptually, the synthesis of target **XylNapN3** was straightforward (Scheme 1B).

Starting from the peracetylated **13**, we deprotected the aromatic hydroxyl group using NH_4_OAc in a ternary mixture of THF:MeOH:H_2_O to give **14** in a good yield. This improved from a previously used methodology for the selective deprotection of aromatic acetyl groups, i.e., KCN in MeOH.^21^ To install the linker and minimize unwanted deprotection of the xylose moiety, we used K_2_CO_3_, followed by acidic desilylation of the crude **15** to give **16** in a good overall yield. The terminal hydroxyl group was then mesylated to give **17**, followed by installation of the azide using microwave heating to generate **18** in an excellent yield. Final deacetylation using K_2_CO_3_ in MeOH furbished the final target **XylNapN3**. The control compounds **XylNap** and **XylNapOH** have been synthesized previously.^5,22^

### Azide-Functionalized Naphthoxylosides as Substrates for β4GalT7

**Figure 2:**
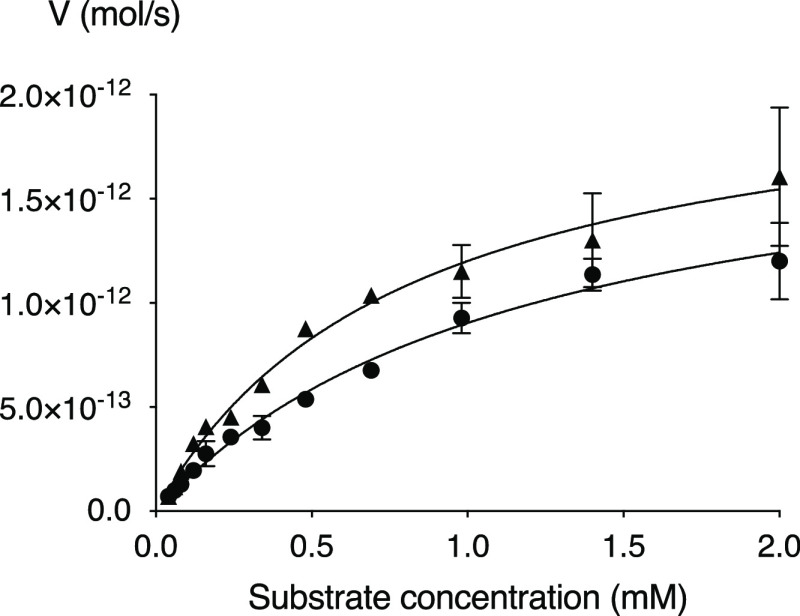
Kinetic profile of **XylNapN3** (filled triangles) compared to that of **XylNap** (filled circles).

To confirm the ability to function as a substrate for β4GalT7, we evaluated **XylNapN3** and compared it to **XylNap** in the galactosylation assay.^23^**XylNapN3** displayed a similar kinetic profile as **XylNap** (Figure 2 and Table 1), suggesting that the azide functionalization does not influence the reaction with β4GalT7.

**Table 1:** Galactosylation by β4GalT7

compound	*K*_m_ (mM)	*V*_max_ (pmol/s)	*k*_cat_ (s^–1^)	*k*_cat_/*K*_m_ (mM^–1^ s^–1^)
**XylNapN3**	0.80	2.17	1.30	1.63
**XylNap**	1.19	1.98	1.19	1.00

### Cellular Uptake and GAG-Priming of Azide-Functionalized Naphthoxylosides

**Figure 3:**
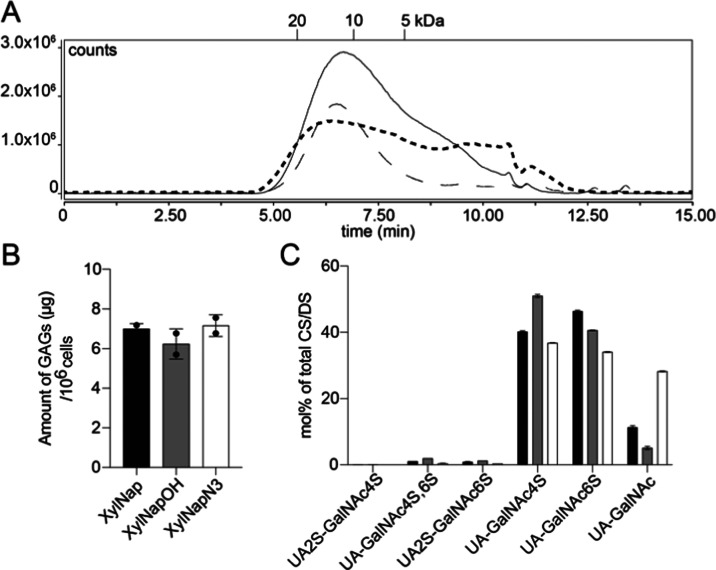
(A) Chromatogram from size-exclusion chromatography on an AdvanceBio SEC column of GAGs from A549 cells treated with **XylNapN3** (dashed bold line), **XylNap** (solid line), and **XylNapOH** (line with long dashes). The indicated molecular weights were obtained using heparin standards. (B) Amount of GAGs primed by A549 cells, as determined by disaccharide analysis. (C) Disaccharide analysis results after the treatment of A549 cells with **XylNap** (black bars), **XylNapOH** (gray bars), or **XylNapN3** (white bars).

To verify that **XylNapN3** is taken up by cells and initiates the biosynthesis of soluble GAGs, A549 cells, i.e., adenocarcinomic human alveolar basal epithelial cells, were treated with **XylNapN3**. The known primers **XylNap** and **XylNapOH** were used as controls. All three compounds were taken up by the cells and functioned as primers of soluble GAG chains excreted to the cell medium. The GAGs were purified by ion-exchange chromatography and analyzed by fluorescence-coupled size-exclusion chromatography (Figure 3a). In comparison to **XylNap** and **XylNapOH**, the treatment of A549 cells with compound **XylNapN3** resulted in greater diversity in the GAG chain length, i.e., slightly longer chains as well as shorter products.

The GAG chains were analyzed for the amount of primed GAGs and disaccharide composition (Figure 3B,C). **XylNapN3** primed a similar amount of GAGs in comparison with **XylNap** and **XylNapOH**. In addition, the disaccharide composition is similar between the three compounds, and the GAGs consisted mainly of disaccharides sulfated in positions 4 and 6 of the GalNAc moieties. However, the treatment of A549 cells with **XylNapN3** resulted in GAGs with a slightly lowered level of sulfation.

### Applications of Azide-Functionalized Naphthoxylosides

**Figure 4:**
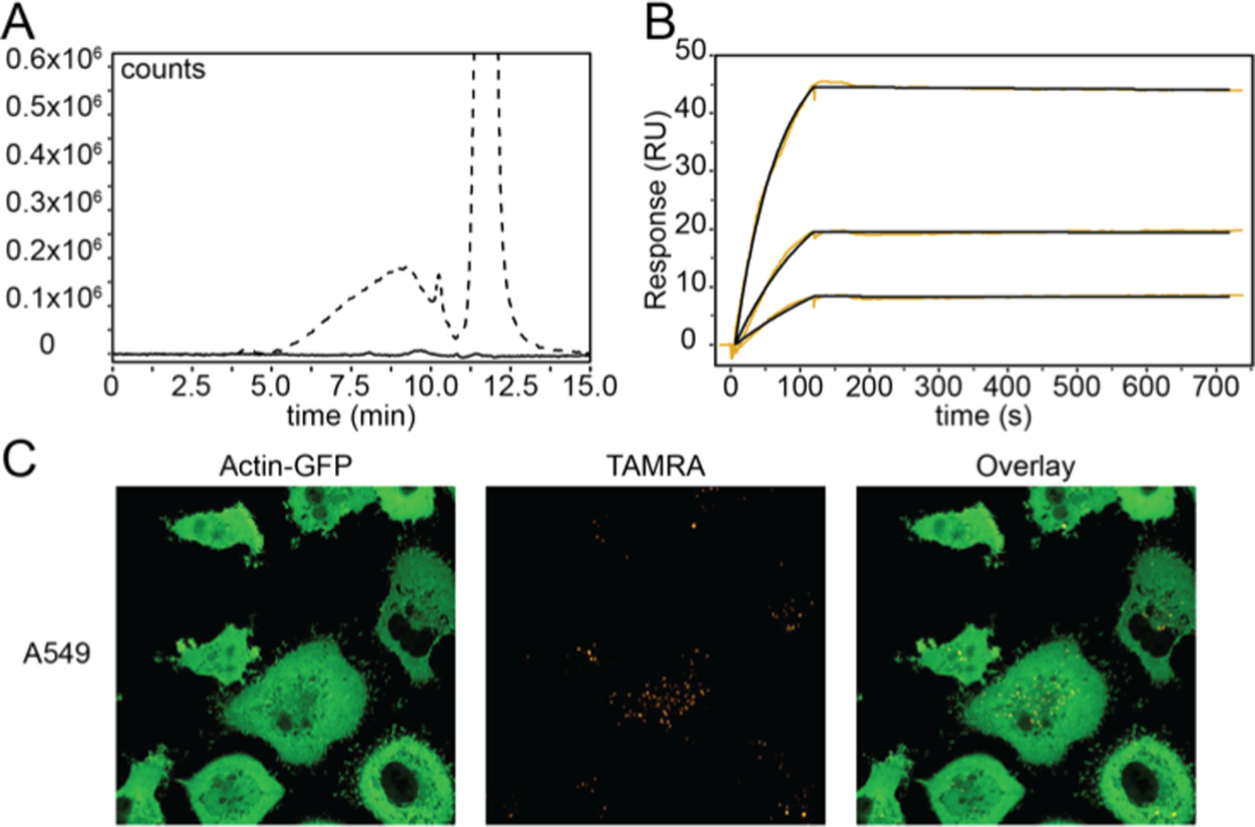
(A) Chromatogram of cell medium before (solid line) and after (dashed line) click reaction between **XylNapN3**-primed GAGs and an Alexa Fluor 647 fluorophore. The broad peak between 5 and 10 min corresponds to xyloside-primed GAGs. The tall peak at 12 min corresponds to the excess fluorophore. Fluorescence was monitored at Ex/Em = 648/671 nm. (B) SPR sensorgram showing the interaction between XylNapN3-primed HS GAGs and hepatocyte growth factor (HGF) at 3, 6, and 12 nM. Black curves show fitting using a 1:1 Langmuir model. (C) Confocal microscopy images of GFP-tagged A549 cells treated with **XylNapN3**–TAMRA conjugates. The compound is efficiently taken up by the cells and localizes to the perinuclear region.

Since most growth media used to cultivate mammalian cells contain high concentrations of serum-derived GAGs, many GAG structure-related experiments are performed in serum-free conditions, which induce unwanted artificial cell responses. We have shown that **XylNapN3** can be taken up by cells and initiate the biosynthesis of soluble GAG chains provided with a handle suitable for functionalization. With such a handle, it is possible to perform experiments in the presence of exogenous GAGs and specifically pull down or derivatize only neosynthesized GAGs. To verify this concept, **XylNapN3**-primed GAGs, isolated from the cell medium by ion-exchange chromatography, were incubated with DBCO-containing Alexa Fluor 647 fluorophore and analyzed by fluorescence-coupled size-exclusion chromatography. The presence of fluorophore-conjugated GAGs was clearly shown, confirming the ability to functionalize the primed GAGs (Figure 4A).

We also labeled **XylNapN3**-primed GAGs, isolated from primary lung fibroblasts, with biotin via the azide handle. Using a streptavidin-coated surface plasmon resonance (SPR) chip, we performed kinetic studies with isolated heparan sulfate (HS) GAGs and the hepatocyte growth factor (HGF) known to interact with HS (Figure 4B). The experiments revealed an association rate (*k*_A_) of 1.31 × 10^6^/(M s) and a dissociation rate (*k*_D_) of 18 × 10^–6^/s, yielding a *K*_D_ of 14 × 10^–12^ between HGF and GAGs isolated from the primary fibroblasts. The χ^2^/ndof residual was calculated to be 0.18 RU^2^.

Finally, **XylNapN3** was labeled with a tetramethylrhodamine (TAMRA) fluorophore, which is known to be efficiently taken up and evenly distributed in living cells.^24^ We treated GFP-tagged A549 cells with the **XylNapN3**–TAMRA conjugate and observed the uptake by confocal microscopy (Figure 4C). The compound localized to the perinuclear region, but no primed GAGs were observed in the conditioned cell medium, in line with what we have observed for **XylPacBlue**.^25^

## Conclusions

In conclusion, we have designed and synthesized a naphthoxyloside, **XylNapN3**, functionalized with an azide moiety. **XylNapN3** was shown to be a substrate for β4GalT7 and was galactosylated with similar kinetics as the known **XylNap**. Furthermore, we have shown that **XylNapN3** is taken up by A549 cells, where it initiated the biosynthesis of soluble GAGs of comparable sizes, and similar disaccharide composition, as GAGs from the known primers **XylNap** and **XylNapOH**. Furthermore, these GAG chains were efficiently reacted in a strain-promoted cycloaddition with a DBCO-containing Alexa Fluor 647 fluorophore. We also showed that **XylNapN3**, labeled with the TAMRA fluorophore, could be used for fluorescence localization microscopy experiments *in vivo*. Finally, GAGs isolated from primary fibroblast stimulated with **XylNapN3** were successfully biotinylated via the azide handle and used in SPR experiments to determine the kinetic parameters for their interaction with the hepatocyte growth factor.

From these data, we argue that **XylNapN3** is a valuable tool for isolating and manipulating cell-specific, soluble GAGs and investigate their interactions with biomolecules, such as growth factors. In addition, we propose that compound **XylNapN3** will be a useful tool for coupling GAGs with, for example, fluorophores, polyhistidine tags, magnetic beads, and SPR sensor chips.

## Experimental Methods

All moisture- and air-sensitive reactions were carried out under an atmosphere of dry nitrogen using oven-dried glassware. All solvents were dried using an MBRAUN SPS-800 Solvent purification system before use unless otherwise stated. Purchased reagents were used without further purification. Chromatographic separations were performed on Matrix silica gel (25–70 μm). Thin-layer chromatography was performed on precoated TLC glass plates with silica gel 60 F_254_ 0.25 mm (Merck). Spots were visualized with UV light or by charring with an ethanolic anisaldehyde solution. Biotage Isolute phase separators were used for drying of combined organic layers. Preparative chromatography was performed on a Biotage Isolera One flash purification system using Biotage SNAP KP-Sil silica cartridges. Optical rotations were measured on a Bellingham and Stanley model ADP450 polarimeter and are reported as [α]_D_^T^ (*c* = g/100 mL), where D indicates the sodium D line (589 nm) and T indicates the temperature. NMR spectra were recorded at ambient temperatures on a Bruker Avance II at 400 MHz (^1^H) and 100 MHz (^13^C) or a Bruker Ascend at 500 MHz (^1^H) and 125 MHz (^13^C) and assigned using 2D methods (COSY, HMQC). Chemical shifts are reported in ppm, with reference to residual solvent peaks (δH CHCl_3_ = 7.26 ppm, CD_3_OH = 3.31 ppm) and solvent signals (δC CDCl_3_ = 77.0 ppm, CD_3_OD = 49.0 ppm). Coupling constant values are given in Hz. Mass spectra were recorded on Waters XEVO G2 (positive ESI). Infrared spectroscopy was recorded on a Bruker α II FT-IR spectrometer. IR was only used to confirm structural features, and only the peak of interest was reported.

The Swedish Research Ethical Committee approved this study in Lund (2008/413, 2011/581), and all experimental protocols were carried out in accordance with the guidelines approved by the ethical committee.

### Synthesis of XylNapN3

#### (6-Hydroxynaphthalen-2-yl) 2,3,4-Tri-*O*-acetyl-β-d-xylopyranoside (**14**)

**13** (1.012 g, 2.20 mmol) was dissolved in THF (20 mL), followed by the addition of MeOH (10 mL) and H_2_O (5 mL) while stirring. After 5 min, NH_4_OAc (2.711 g, 35.17 mmol) was introduced, and the mixture was heated to 40 °C and left overnight. Upon completion, the reaction was diluted with H_2_O and extracted with DCM (4×). The organic phase was washed with NaCl (sat. aq), dried, and concentrated in vacuo. The residue was purified by flash chromatography (heptane:EtOAc, 10–50%) to yield **14** (774 mg, 1.85 mmol, 84%) as an amorphous solid. The analysis was in accordance with the published data.^21^

#### (6-(3-Hydroxypropoxy)naphthalen-2-yl) 2,3,4-Tri-*O*-acetyl-β-d-xylopyranoside (**16**)

**14** (651 mg, 1.56 mmol) was dissolved in DMF (8 mL) under an Ar(g) atmosphere while stirring, followed by 3-(*tert*-butyldimethylsilyloxy)propyl bromide^26^ (524 mg, 2.07 mmol). K_2_CO_3_ (475 mg, 3.44 mmol) was introduced, and the mixture was heated to 40 °C and left overnight. Upon completion, the heating was removed and H_2_O was added. The aqueous phase was extracted with DCM (4×). The organic phase was then dried and coevaporated twice with toluene in vacuo. Crude **15** (933 mg) was then dissolved in MeOH (29.2 mL), followed by HCl (37%, 810 μL) while stirring. Upon completion after 30 min, the mixture was neutralized and concentrated in vacuo. The crude residue was purified by flash chromatography (SiO_2_, heptane:EtOAc, 33–100%) to yield **16** (467 mg, 0.98 mmol, 63% over two steps) as an oily residue. [a]_D_^25^ −2.2° (*c* 0.46, CDCl_3_). ^1^H NMR (400 MHz, CDCl_3_) δ 7.65 (dd, *J* = 9.3, 4.6 Hz, 2H), 7.32 (d, *J* = 2.5 Hz, 1H), 7.17–7.12 (m, 3H), 5.29–5.20 (m, 3H), 5.04 (td, *J* = 7.5, 4.8 Hz, 1H), 4.27 (dd, *J* = 12.1, 4.7 Hz, 1H), 4.23 (t, *J* = 6.0 Hz, 2H), 3.91 (q, *J* = 5.5 Hz, 2H), 3.58 (dd, *J* = 12.1, 7.6 Hz, 1H), 2.15–2.07 (m, 11H).^13^C NMR (101 MHz, CDCl_3_) δ 170.12, 170.01, 169.58, 156.09, 153.06, 131.16, 129.59, 128.75, 128.48, 119.62, 119.41, 111.97, 106.98, 99.05, 70.85, 70.37, 68.69, 65.94, 62.05, 60.71, 32.14, 20.94, 20.92, 20.89. HRMS (*m*/*z*): [M]^+^ calcd for C_24_H_28_O_10_Na: 499.1580; found: 499.1582.

#### (6-(3-((Methylsulfonyl)oxy)propoxy)naphthalen-2-yl) 2,3,4-Tri-*O*-acetyl-β-d-xylopyranoside (**17**)

**16** (267 mg, 0.56 mmol) was dissolved in pyridine (5.6 mL) while stirring and cooled to 0 °C. Methanesulfonyl chloride (174 μL, 2.24 mmol) was then slowly added dropwise. After 20 min, the reaction was allowed to reach r.t. Upon completion after 1 h, the reaction was diluted with H_2_O and DCM. The aqueous phase was extracted with DCM (4×). The organic phase was then dried and concentrated in vacuo. The crude residue was purified by flash chromatography (SiO_2_, heptane:EtOAc, 33–100%) to yield **17** (275 mg, 0.50 mmol, 89%) as an oily residue. [a]_D_^25^ −21,4° (*c* 1.0, CDCl_3_). ^1^H NMR (400 MHz, CDCl_3_) δ 7.65 (dd, *J* = 8.8, 4.3 Hz, 2H, Ar-H), 7.32 (d, *J* = 2.5 Hz, 1H, Ar-H), 7.20–7.09 (m, 3H, Ar-H), 5.31–5.18 (m, 3H, H-1, H-2, H-3), 5.04 (td, *J* = 7.3, 4.7 Hz, 1H, H-4), 4.49 (t, *J* = 6.1 Hz, 2H, -CH_2_OMs), 4.27 (dd, *J* = 12.1, 4.7 Hz, 1H, H-5), 4.19 (t, *J* = 5.8 Hz, 2H, -OCH_2_-), 3.58 (dd, *J* = 12.1, 7.6 Hz, 1H, H-5), 3.00 (s, 3H, SO_2_CH_3_), 2.29 (p, *J* = 6.0 Hz, 2H, -CH_2_-), 2.13–2.07 (m, 9H, 3× COCH_3_). ^13^C NMR (101 MHz, CDCl_3_) δ 170.10, 170.00, 169.56, 155.79, 153.15, 131.08, 129.69, 128.89, 128.50, 119.51, 119.45, 111.97, 106.99, 99.00, 70.81, 70.34, 68.67, 66.90, 63.40, 62.04, 37.42, 29.27, 20.93, 20.91, 20.89. HRMS (*m*/*z*): [M]^+^ calcd for C_25_H_30_O_12_SNa: 577.1346; found: 577.1360.

#### (6-(3-Azidopropoxy)naphthalen-2-yl) 2,3,4-Tri-*O*-acetyl-β-d-xylopyranoside (**18**)

**17** (275 mg, 0.50 mmol) was dissolved in DMF (4 mL), followed by NaN_3_ (65 mg, 0.99 mmol). The mixture was then heated in a microwave reactor for 30 min at 90 °C. Upon completion, the reaction was diluted with H_2_O and DCM. The aqueous phase was extracted with DCM (4×). The organic phase was then dried and coevaporated twice with toluene in vacuo. The crude residue was purified by flash chromatography (SiO_2_, heptane:EtOAc, 33–100%) to yield **18** (220 mg, 0.44 mmol, 88%) as an oily residue. [a]_D_^25^ −13.1° (*c* 1.0, CDCl_3_). IR 2099 cm^–1^ (N_3_). ^1^H NMR (400 MHz, CDCl_3_) δ 7.65 (dd, *J* = 8.9, 4.1 Hz, 2H, Ar-H), 7.32 (d, *J* = 2.5 Hz, 1H, Ar-H), 7.19–7.09 (m, 3H, Ar-H), 5.34–5.19 (m, 3H, H-1, H-2, H-3), 5.04 (td, *J* = 7.1, 4.7 Hz, 1H, H-4), 4.27 (dd, *J* = 12.1, 4.7 Hz, 1H, H-5), 4.15 (t, *J* = 6.0 Hz, 2H, -CH_2_O-), 3.57 (dd, *J* = 12.1, 7.6 Hz, 1H, H-5), 3.56 (t, *J* = 6.6 Hz, 2H, -CH_2_-OH), 2.11 (p, *J* = 6.2 Hz, 2H, -CH_2_-), 2.11–2.09 (m, 9H, 3xCOCH_3_).^13^C NMR (101 MHz, CDCl_3_) δ 170.11, 170.00, 169.56, 155.99, 153.09, 131.13, 129.63, 128.79, 128.48, 119.60, 119.43, 112.00, 106.96, 99.05, 70.85, 70.37, 68.69, 64.72, 62.05, 48.46, 28.94, 20.93, 20.91, 20.89. HRMS (*m*/*z*): [M]^+^ calcd for C_24_H_27_N_3_O_9_Na: 524.1645; found: 524.1637.

#### (6-(3-Azidopropoxy)naphthalen-2-yl) β-d-Xylopyranoside (**XylNapN3**)

**18** (216 mg, 0.43 mmol) was dissolved in MeOH (10 mL), followed by the addition of K_2_CO_3_ (357 mg, 2.58 mmol). Upon completion after 1.5 h, the reaction was acidified, and the solvent was removed. The crude mixture was then recrystallized from H_2_O and filtered, yielding **XylNapN3** (137 mg, 0.36 mmol, 84%) as a white solid. m.p.: 147–151 °C. [a]_D_^25^ −22.8° (*c* 0.57, MeOD_4_). IR 2098 cm^–1^ (N_3_). ^1^H NMR (400 MHz, MeOD) δ 7.68 (t, *J* = 8.7 Hz, 2H, Ar-H), 7.37 (d, *J* = 2.5 Hz, 1H, Ar-H), 7.27–7.20 (m, 2H, Ar-H), 7.12 (dd, *J* = 8.9, 2.5 Hz, 1H, Ar-H), 4.97 (d, *J* = 7.1 Hz, 2H, H-1), 4.15 (t, *J* = 6.0 Hz, 2H, -OCH_2_-), 3.96 (dd, *J* = 11.3, 5.2 Hz, 1H, H-5), 3.65–3.38 (m, 6H, H-4, H-3, H-2, H-5′, -CH_2_N_3_), 2.09 (p, *J* = 6.4 Hz, 2H, -CH_2_-). ^13^C NMR (101 MHz, MeOD) δ 157.17, 155.22, 132.30, 131.10, 129.57, 129.18, 120.41, 120.26, 112.44, 107.85, 103.26, 77.76, 74.83, 71.08, 66.97, 65.89, 48.98,^1^ 29.87. HRMS (*m*/*z*): [M]^+^ calcd for C_18_H_21_N_3_O_6_(HCOO^–^): 420.1407; found: 420.1400.

### β4GalT7 Assay

The β4GalT7 enzymatic assay was performed as previously described.^27^ Briefly, β4GalT7 (50 ng) was mixed in 96-well polypropylene plates with UDP-Gal (1 mM final concentration) and various concentrations of xylosides in a final volume of 50 μL MES buffer (20 mM, pH 6.2) supplemented with MnCl_2_ (10 mM). Incubation was performed at 37 °C for 30 min, and the reaction was stopped by cooling at 4 °C and the addition of HPLC eluent (70% NH_4_OAc (60 mM, pH 5.6)–30% CH_3_CN (v/v)) before HPLC analysis.

### Xyloside Stimulation of A549 Cells

Actin-EmGFP-modified A549 cells were prepared as previously described (REF XPB paper) and grown to approx. 70% confluence in DMEM/F-12/GlutaMAX (Thermo Fisher Scientific) supplemented with 10% FBS (Thermo Fisher Scientific), 100 units/mL penicillin, and 100 μg/mL streptomycin (Sigma-Aldrich). A stock solution of **XylNapN3** was prepared at 50 mM in DMSO and added to the cells in OptiPRO SFM medium (Thermo Fisher Scientific) at a concentration of 100 μm. After 24 h of treatment, medium samples were either analyzed directly by fluorescence detection size-exclusion chromatography (FSEC) on an AdvanceBio SEC column (Agilent).

### Glycosaminoglycan Purification

Glycosaminoglycans (GAGs) secreted into the cell medium were purified by anion-exchange chromatography, as described below. After supplementation with the Triton X-100 detergent (Sigma-Aldrich) to 0.1%, the medium was protease-treated with 0.5 mg/mL Pronase (Sigma-Aldrich) for 16 h at 50 °C, after which the protease was heat-inactivated at 95 °C for 10 min. To each sample, MgCl_2_ was added to 2 mM to aid degradation of nucleic acids with 50 U/mL turbonuclease (Sigma-Aldrich) for 1 h at 37 °C. To facilitate the specific binding of GAGs to the DEAE-Sephacel ion-exchange resin, samples were buffered to pH 5.4 using a concentrated sodium acetate solution for a final concentration of 100 mM. Each 10 mL sample was then purified on a 100 v bed of DEAE-Sephacel (Cytiva Life Sciences) packed in 2 mL Pierce disposable columns (Thermo Fisher Scientific). The resin was washed twice with a 500 μL solution of sodium acetate (20 mM), NaCl (100 mM), and Triton X-100 (0.1%) and twice with a 500 μL solution of the same buffer without detergent added. GAGs were eluted using 500 μL of a 2 M solution of NaCl and precipitated overnight at −20 °C after the addition (3:1 v/v) of ethanol (>95%) saturated with sodium acetate. GAGs were pelleted by centrifugation at 20 000*g*, 4 °C, for 10 min, washed with 500 μL cold ethanol (>95%), and once again pelleted by centrifugation before being dried in a SpeedVac vacuum concentrator (Thermo Fisher Scientific).

### Disaccharide Analysis

Disaccharide analysis of xyloside-primed GAGs from A549 cells was essentially performed as previously described.^28^ Briefly, purified GAGs corresponding to 10% of the total medium sample from a T75 cell culture flask were split into two samples and treated for 6 h at 37 °C with 10 mIU/sample chondroitinase ABC (Sigma-Aldrich) and 20 + 20 mIU/sample heparinase II + III (prepared in-house), respectively, in 10 μL ammonium acetate buffer (50 mM, pH 7.1). After GAG degradation, disaccharides were fluorescently labeled by reductive amination using 10 μL 2-aminoacridone (20 mM in acetic acid/DMSO 15/85, v/v, pH around 4–5) mixed with sodium cyanoborohydride (1 M final concentration). Labeled disaccharides were separated by reversed-phase HPLC, detected with a fluorescence detector, and quantified using labeled disaccharide standards (Iduron) of known concentration.

### Glycosaminoglycan Biotinylation

For interaction studies between xyloside-primed GAGs and the growth factor HGF, primary lung fibroblast isolated from the distal lung tissue from a healthy subject was grown in DMEM/F-12/GlutaMAX (Thermo Fisher Scientific) supplemented with 10% FBS (Thermo Fisher Scientific), 100 units/mL penicillin, and 100 μg/mL streptomycin (Sigma-Aldrich). A stock solution of **XylNapN3** was prepared at 50 mM in DMSO and added to the cells at 100 μM. Cells were stimulated for 24 h, after which the GAGs were purified as above. Purified GAGs from 10 mL conditioned medium (from a T75 flask) were reacted with Click-iT biotin sDIBO alkyne (Thermo Fisher Scientific) at a final concentration of 0.2 mg/mL in DPBS. Excess biotinylation reagent was removed using Amicon Ultra 10 kDa cutoff centrifugal filters (Merck Millipore).

### Surface Plasmon Resonance Interaction between Glycosaminoglycans and Growth Factors

The GAG–biotin complexes were immobilized on a 30 nm streptavidin-derivatized linear polycarboxylate hydrogel with medium charge density (SPSM SAHC30M, Xantec). The surface was conditioned with 50 mM NaOH and 1 M NaCl, followed by running buffer until a stable baseline. Then, the ligand surface was immobilized at a flow rate of 10 μL/min for 1 min with HS GAGs, corresponding to ∼60% isolated chondroitinase ABC-treated GAGs from a T75 cell culture plate, in running buffer (PBS with 0.01% Tween 20) for a total RU of 93. To minimize unspecific binding, Click-iT biotin sDIBO alkyne (Thermo Fisher Scientific) was bound to free streptavidin sites by running 10 μg/mL for 1 min on both the channel with GAGs and the reference. The treatment was repeated once more to make sure all streptavidin sites were blocked. For the runs with HGF (R&D Systems), the protein was dissolved in running buffer and diluted in incremental steps of 2× (12.22, 6.11, and 3.05 nM final concentration). Injections were started at the lowest concentration with a 2 min association time, a 10 min dissociation time, and 2 min of regeneration repeated for all concentrations. Analysis and curve fitting were done in Sierra Analyzer 3.1.36 (Bruker), with the reference spot and the blank subtracted from each graph.

### DBCO–Alexa Fluor 647 and DBCO–TAMRA Labeling

For labeling with the TAMRA fluorophore, **XylNapN3** was dissolved in Dulbeccoʼs phosphate-buffered saline (DPBS) at a concentration of 1.5 mM (from 50 mM stock in DMSO). Approximately 20 μg of lyophilized dibenzylcyclooctyne-PEG4-5/6-tetramethylrhodamine (Jena Bioscience) was added to the xyloside sample, and the sample was incubated for 1 h. The labeled xyloside was purified by reversed-phase chromatography before treatment of cells as described above.

A sample with **XylNapN3**-primed GAGs was labeled in the same manner as above but instead with dibenzylcyclooctyne–Alexa Fluor 647 (Jena Bioscience). Labeled GAGs were purified using Amicon Ultra 10 kDa cutoff centrifugal filters (Merck Millipore) and analyzed using FSEC (Abs/Em = 648/671 nm) on an AdvanceBio SEC column (Agilent).

### Confocal Microscopy of A549 Cells

Localization experiments were performed on a Nikon Ti2 microscope equipped with a Crest X-Light V3 spinning disc unit. Images were captured using a 100× Plan Apo Lambda NA 1.45 objective with cells seeded on Lab-Tek 8-well (0.8 cm^2^) borosilicate (0.17 mm) slides (Thermo Fisher Scientific). The cell layer was washed twice with Dulbecco’s phosphate-buffered saline (Sigma-Aldrich) and then covered with an OptiPRO SFM medium before imaging. TAMRA-labeled xyloside (and its products) was detected at Abs/Em = 560/565 nm.

## Supporting Information Available

Synthesis and characterization of nonevaluated xylosides, and ^1^H- and ^13^C NMR of synthesized compounds (PDF)

## Footnotes

1 Inferred from 2D ^1^H–^13^C HMQC experiment.
