# Supplementary material for: Azide-Functionalized Naphthoxyloside as a Tool for Glycosaminoglycan Investigations
Source: Bioconjug Chem. 2021 Nov 16;32(12):2507–15. doi: 10.1021/acs.bioconjchem.1c00473 (PMC8678990; doi:10.1021/acs.bioconjchem.1c00473)
Supplement: Supplementary file 1 — bc1c00473_si_001.pdf [file bc1c00473_si_001.pdf]

Supporting information for

An azide functionalized naphthoxyloside as a tool for glycosaminoglycan investigations.

Daniel Willén,† Roberto Mastio,† Zackarias Söderlund,‡ Sophie Manner,† Gunilla Westergren-Thorsson,‡ Emil Tykesson, ‡ Ulf Ellervik†‡*

† Centre for Analysis and Synthesis, Centre for Chemistry and Chemical Engineering, Lund University, P.O. Box 124, SE-221 00 Lund, Sweden

‡ Department of Experimental Medical Science, Lund University, P.O. Box 117, SE-221 00 Lund, Sweden.

* Centre for Analysis and Synthesis, Centre for Chemistry and Chemical Engineering, Lund University, P.O. Box 124, SE-221 00 Lund, Sweden. E-mail: ulf.ellervik@chem.lu.se.

1. **Synthesis of non-evaluated naphthoxylosides** **S2-S5**
2. **^{1}H- and ^{13}C-NMR of synthesized compounds** **S6-S20**
3. **References** **S21**

Synthesis of non-evaluated naphthoxylosides

6-(bromomethyl)naphthalen-2-yl acetate *(2)*. PBr_3 (95 µL, 0.99 mmol) was added to a stirred solution of 6-(hydroxymethyl)naphthalen-2-yl acetate[1] **1** (214 mg, 0.99 mmol) in CH_2Cl_2 (8 mL) at 0 ºC. After 1.5 h, H_2O (5 mL) was added and the reaction mixture was extracted with CH_2Cl_2 (3x5 mL). The combined organic phases were dried before removal of solvent under reduced pressure. The crude residue was purified by column chromatography (SiO_2, Petroleum ether: pentane, 98:2) to yield **2** (155 mg, 0.56 mmol, 56%) as an amorphous white solid. ^1H NMR (400 MHz, $CDCl_3$) δ 7.84-7.78 (m, 3H, Ar-H), 7.56-7.51 (m, 2H, Ar-H), 7.25 (dd, J = 8.8, 2.8 Hz, 1H, Ar-H), 4.66 (s, 3H, $-CH_2$-Br), 2.36 (2, 3H, Ac). ^{13}C NMR (101 MHz, $CDCl_3$) δ 169.69, 149.05, 135.21, 133.57, 131.30, 129.60, 128.65, 127.85, 127.69, 121.94, 118.66, 33.96, 21.35.

6-(azidomethyl)naphthalen-2-yl acetate *(3)*. *N*aN_3 (91 mg, 1.41 mmol) was added to a stirred solution of **2** (155 mg, 0.55 mmol) in DMSO (2 mL) at 0 ºC. After 2 h, H_2O (5 mL) was added and the reaction mixture was extracted with EtOAc (3x5 mL). The combined organic phases were washed with brine (10 mL) and dried before removal of the solvent under vacuum. The crude residue was purified by column chromatography (SiO_2, Petroleum ether: pentane, 95% to 85%) to yield **3** (119 mg, 0.49 mmol, 89 %) as an amorphous white solid. ^1H NMR (400 MHz, $CDCl_3$) δ 7.84 (dd, J = 12.8, 8.8 Hz, 2H, Ar-H), 7.77 (s, 1H, Ar-H), 7.58 (d, J = 2.0 Hz, 1H, Ar-H), 7.44 (dd, J = 8.8, 2.0 Hz, 1H, Ar-H), 7.27 (dd, J = 9.2, 2.4 Hz, 1H, Ar-H), 4.49 (s, 2H, $-CH_2-N_3$). ^{13}C NMR (101 MHz, $CDCl_3$) δ 169.70, 148.82, 133.51, 132.86, 131.33, 129.55, 128.60, 127.13, 126.72, 121.89, 118.61, 54.95, 21.30. IR 2094 cm^{-1} (N_3).

6-(azidomethyl)naphthalen-2-ol *(4)*. 1M NaOMe (50 µL, 0.05 mmol) was added to a stirred solution of **3** (32 mg, 0.13 mmol) in MeOH (1 mL) at r.t. After 2 h, Amberlite IR-120H^+ was added until neutral pH. The reaction mixture was filtered before removal of the solvent in vacuo. The crude residue was purified by column chromatography (SiO_2, Petroleum ether: pentane, 9:1) to yield **4** (19 mg, 0.10 mmol, 73 %) as an amorphous white solid. ^1H NMR (400 MHz, $CDCl_3$) δ 7.75 (d, J = 8.8 Hz, 1H, Ar-H), 7.70 (s, 1H, Ar-H), 7.69 (d, J = 8.8 Hz, 1H, Ar-H), 7.38 (dd, J = 8.4, 2.0 Hz, 1H, Ar-H), 7-15-7.12 (m, 2H, Ar-H), 5.30 (s, 1H, OH), 4.46 (s, 2H, $-CH_2-N_3$). ^{13}C NMR (101 MHz, $CDCl_3$) δ 153.90, 134.42, 130.57, 130.05, 128.75, 127.40, 127.37, 126.81, 118.44, 109.63, 55.15.

(6-(2-azidomethyl)naphthalen-2-yl) β-D-xylopyranoside *(5)*. $BF_3 \cdot OEt_2$ (38 µL, 0.30 mmol) was added to a stirred solution of Et_3N (8 µL, 0.06 mmol), **4** (32 mg, 0.16 mmol), and peracetylated xylose (38 mg, 0.12 mmol) in CH_2Cl_2 (0.5 mL) at 0 ºC. After 5 h, saturated aqueous $NaHCO_3$ (5 mL) was added and the reaction mixture was extracted with CH_2Cl_2 (3x5 mL) and EtOAc (1x5 mL). The combined organic phases were dried before removal of solvent under reduced pressure. The crude mixture was dissolved in MeOH (1 mL) before addition of 1M NaOMe (30 µL, 0.03 mmol). After 1 h, Amberlite IR-120H^+ was added until neutral pH. The reaction mixture was filtered before removal of the solvent in vacuo. The crude residue was purified by column chromatography (SiO_2, CH_2Cl_2: MeOH, 98:2) to yield **5** (8 mg, 0.02 mmol, 13 % starting from **4**) as an amorphous white solid. ^1H NMR (400 MHz, MeOD) δ 7.82-

7.80 (m, 2H, Ar-H), 7.77 (s, 1H, Ar-H), 7.44-7.41 (m, 2H, Ar-H), 7.30 (dd, *J* = 8.8, 2.4 Hz, 1H, Ar-H), 5.04 (d, *H* = 7.2 Hz, 1H, H-5), 4.49 (s, 2H, -CH_2-N_3), 3.98 (dd, *J* = 11.6, 5.6 hz, 1H, H-5), 3.64-3.58 (m, 1H, H-4), 3.53-3.43 (m, 3H, H-2, H-3, H-5). ^{13}C NMR (101 MHz, MeOD) δ 147.60, 126.01, 123.48, 121.49, 121.00, 119.42, 118.70. 118.27, 111.05, 102.28, 93.33, 68.25, 65.31, 61.57, 57.53, 46.27. IR 2089 cm^{-1} (N_3).

2-(6-acetoxynaphthalen-2-yl)acetic acid *(7)*. Acetic anhydride (30 mL, 317 mmol) was added to a stirred solution of 2-(6-hydroxynaphthalen-2-yl)acetic acid **6** (5 g, 24.7 mol) in dry pyridine (45 mL) at r.t. After 24 h, the solvent was removed under reduced pressure. The crude residue was co-eavaporated with toluene several times. The crude residue was purified by column chromatography (SiO_2, CH_2Cl_2: MeOH, 98:2) to yield **7** (4.08 g, 16.7 mmol, 68 %) as a brown grey solid. ^{1}H NMR (400 MHz, DMSO-d_6) δ 7.91 (d, *J* = 8.9 Hz, 1H, Ar-H), 7.85 (d, *J* = 8.5 Hz, 1H, Ar-H), 7.74 (s, 1H, Ar-H), 7.64 (d, *J* = 2.3 Hz, 1H, Ar-H), 7.37 (dd, *J* = 8.4, 1.7 Hz, 1H, Ar-H), 7.29 (dd, *J* = 8.8, 2.4 Hz, 1H, Ar-H), 3.94 (s, 2H, -CH_2-), 2.32 (s, 3H, Ac). ^{13}C NMR (101 MHz, DMSO-d_6) δ 206.03, 169.44, 147.99, 132.60, 132.11, 131.0, 128.9 (2C), 127.89, 127.38, 121.78, 118.34, 49.61f, 20.92.

6-(2-hydroxyethyl)naphthalen-2-yl acetate *(8)*. 2M BH_3·THF (8 mL, 16.4 mmol) was added dropwise to a stirred solution of **7** (4.08 g, 16.7 mmol) in dry THF (40 mL) at 0 ºC. After 2 h at r.t., more 2M BH_3·THF (6 mL, 12.0 mmol) was added. After 2 h, the temperature was lowered to 0 ºC where after 0.5 M HCl (20 mL) was added dropwise. After 20 h at r.t., the reaction mixture was extracted with diethyl ether (3x 25 mL). The combined organic phases were washed with sat. aqueous $NaHCO_3$ (50 mL) and dried before removal of the solvent under reduced pressure. The crude residue was purified by column chromatography (SiO_2, petroleum ether:diethyl ether, 4:6) to yield **8** (2.63 g, 11.4 mmol, 68 %) as an amorphous yellow solid. ^{1}H NMR (400 MHz, $CDCl_3$) δ 7.79 (d, *J* = 8.8 Hz, 1H, Ar-H), 7.74 (d, *J* = 8.4 Hz, 1H, Ar-H), 7.65 (s, 1H, Ar-H), 7.53 (d, *J* = 2.2 Hz, 1H, Ar-H), 7.35 (dd, *J* = 8.4, 1.8 Hz, 1H, Ar-H), 7.22 (dd, *J* = 8.9, 2.4 Hz, 1H, Ar-H), 3.89 (t, *J* = 6.6 Hz, 2H, -CH_2-OH), 2.99 (t, *J* = 6.6 Hz, 2H, Ar-CH_2-), 2.35 (s, 3H, Ac). ^{13}C NMR (101 MHz, $CDCl_3$) δ 169.84, 148.11, 136.09, 132.56, 131.65, 129.04, 128.26, 128.00, 127.41, 121.40, 118.41, 63.51, 39.27, 21.28.

6-(2-((methylsulfonyl)oxy)ethyl)naphthalen-2-yl acetate *(9)*. Methanesulfonyl chloride (109 µL, 1.41 mmol) was added to a stirred solution of 6-(2-hydroxyethyl)naphthalen-2-yl acetate[1] **8** (217 mg, 0.94 mmol) in pyridine (5 mL) at 0 ºC. After 3 h, H_2O (4 mL) was added and the aqueous phase were extracted with diethyl ether (3x5 mL). The combined organic phases were washed with saturated aqueous $NaHCO_3$ (10 mL), aqueous saturated NH_4Cl (10 mL), and brine (10 mL). The organic phase was dried and concentrated in vacuo. The crude residue was purified by column chromatography (SiO_2, Heptane: EtOAc, 9:1) to yield **9** (231 mg, 0.75 mmol, 79%) as an amorphous yellow solid. ^{1}H NMR (400 MHz, $CDCl_3$) δ 7.81 (d, *J* = 8,8 Hz, 1H, Ar-H), 7.78 (d, *J* = 8.4 Hz, 1H, Ar-H) 7.70 (s, 1H, Ar-H), 7.54 (dd, J = 2.0 Hz, 1H, Ar-H), 7.37 (dd, *J* = 8.4, 1.6 Hz, 1H, Ar-H), 7.24 (dd, *J* = 8.8, 2.4 Hz, 1H, Ar-H) 4.50 (t, *J* = 6.8 Hz, 2H, -CH_2-), 3.22 (t, *J* = 3.22 Hz, 2H, -CH_2-N_3), 2.85 (s, 3H, CH_3-) 2.36 (s, 3H, CH_3-). ^{13}C NMR (101 MHz, $CDCl_3$) δ 169.80. 148.49, 133.87, 132.89, 131.66, 129.21, 128.35, 127.91, 127.74, 121.78, 118.57, 70.15, 37.57, 35.86, 21.36.

6-(2-azidoethyl)naphthalen-2-yl acetate *(10)*. NaN_3 (98 mg, 1.51 mmol) was added to a stirred solution of **9** (233 mg, 0.76 mmol) in DMF (2 mL) at 0 ºC and the temperature was let to r.t. After 5 h, another portion of NaN_3 (50 mg, 1.51 mmol) was added. After 24 h, H_2O (5 mL) was added and the reaction mixture was extacted with EtOAc (3x5 mL). The combined organic phases were washed with brine (10 mL) and dried before removal of the solvent under vacuum. The crude residue was purified by column chromatography (SiO_2, Heptane: EtOAc, 9:1) to yield **10** (143 mg, 0.56 mmol, 74 %) as an amorphous white solid. ^1H NMR (400 MHz, $CDCl_3$) δ 8.80 (d, *J* = 8.8 Hz, 1H, Ar-H) 7.77 (d, *J* = 8.4 Hz, 1H, Ar-H), 7.57 (s, 1H, Ar-H), 7.56 (d, *J* = 2.4 Hz, 1H, Ar-H), 7.35 (dd, *J* = 8.4, 1.6 Hz, 1H, Ar-H), 7.25 (dd, *J* = 8.8, 2.4 Hz, 1H, Ar-H), 3.58 (t, *J* = 7.2 Hz, 2H, $-CH_2-$), 3.18 (t, *J* = 7.2 Hz, 2H, $-CH_2-N_3$). ^{13}C NMR (101 MHz, $CDCl_3$) δ 169.68, 148.27, 135.52, 132.68, 131.61, 129.08, 128.09, 127.80, 127.24, 121.52, 118.43, 52.34, 35.42, 21.24.

6-(2-azidoethyl)naphthalen-2-ol *(11)*. 1M NaOMe (50 µL, 0.05 mmol) was added to a stirred solution of **10** (140 mg, 0.55 mmol) in MeOH (1 mL) at r.t. After 2 h, Amberlite IR-120H^+ was added until neutral pH. The reaction mixture was filtered before removal of the solvent in vacuo. The crude residue was purified by column chromatography (SiO_2, Heptane: EtOAc, 8:2) to yield **11** (105 mg, 0.49 mmol, 89 %) as an amorphous white solid. ^1H NMR (400 MHz, $CDCl_3$) δ 7.71 (d, *J* = 8.8 Hz, 1H, Ar-H), 7.65 (d, *J* = 8.4 Hz, 1H, Ar-H), 7.60 (s, 1H, Ar-H), 7.30 (dd, *J* = 8.4, 1.6 Hz, 1H, Ar-H), 7.13 (d, *J* = 2.4 Hz, 1H, Ar-H), 7.10 (dd, *J* = 8.4, 2.4 Hz, 1H, Ar-H), 3.58 (t, *J* = 7.2 Hz, 2H, $-CH_2-$), 3.02 (t, *J* = 7.2 Hz, 2H, $-CH_2-N_3$). ^{13}C NMR (101 MHz, $CDCl_3$) δ 153.35, 133.59, 133.30, 129.62, 129.17, 127.83, 127.36, 126.94, 118.16, 109.52, 52.61, 35.46.

(6-(2-azidoethyl)naphthalen-2-yl) 2,3,4-tri-*O*-acetyl-β-D-xylopyranoside *(19)*. $BF_3{\cdot}OEt_2$ (117 µL, 0.93 mmol) was added to a stirred solution of Et_3N (26 µL, 0.19 mmol), **11** (102 mg, 0.48 mmol), and peracetylated xylose (118 mg, 0.37 mmol) in CH_2Cl_2 (2 mL) at 0 ºC. After 20 h at r.t, saturated aqueous $NaHCO_3$ (5 mL) was added and the reaction mixture was extracted with DCM (3x5 mL). The combined organic phases were dried before removal of solvent under reduced pressure. The crude residue was purified by column chromatography (SiO_2, Toluen: EtOAc, 9:1) to yield **19** (31 mg, 0.07 mmol, 18 %) as a clear oil. ^1H NMR (400 MHz, $CDCl_3$) δ 7.74-7.70 (m, 2H, Ar-H), 7.62 (s, 1H, Ar-H), 7.35-7.31 (m, 2H, Ar-H), 7.18 (dd, *J* = 8.8, 2.4 Hz, 1H, Ar-H), 5.28 (d, *J* = 5.6 Hz, 1H, H-1), 5.29-5.21 (m, 2H, H2, H-3), 5.07-5.02 (m, 1H, H-4), 4.27 (dd, *J* = 12.4, 4.8 Hz, 1H, H-5), 3.62-3.56 (m, 3H, H-5, $-CH_2-$), 3.03 (t, *J* = 7.2 Hz, 2H, $-CH_2-N_3$). ^{13}C NMR (101 MHz, $CDCl_3$) δ 170.08, 169.99, 169.55, 154.33, 134.38, 133.14, 130.24, 129.41, 127.87, 127.69, 127.26, 119.22, 111.33, 98.65, 70.66, 70.20, 68.58, 61.96, 52.50, 35.46, 20.91, 20.89, 20.86.

(6-(2-azidoethyl)naphthalen-2-yl) β-D-xylopyranoside *(12)*. 1M NaOMe (50 µL, 0.05 mmol) was added to a stirred solution of **19** (52 mg, 0.11 mmol) in MeOH (1 mL) at r.t. After 2 h, Amberlite IR 120 H^+ was added until neutral pH. The reaction mixture was filtered before removal of solvent under reduced pressure. The crude residue was purified by column chromatography (SiO_2, CH_2Cl_2: MeOH, 98:2) to yield **12** (34 mg, 0.10 mmol, 90 %) as an amorphous white solid. ^1H NMR (400 MHz, MeOD) δ 7.74 (d, J = 8.4 Hz, 1H, Ar-H), 7.72 (d, J = 8.0 Hz, 1H, Ar-H), 7.64 (s, 1H, Ar-H), 7.39 (d, J = 2.4 Hz, 1H, Ar-H), 7.34 (dd, J = 8.4, 1.6 Hz, 1H, Ar-H), 7.25 (dd J = 9.2, 2.4 Hz, 1H, Ar-H), 5.01 (d, J = 8.0 Hz, 1H, H-1), 3.97 (dd, J = 11.2, 5.2 Hz, 1H, H-5), 3.64-3.41 (m, 6H, H-2, H-3, H-4, H-5, -CH_2-), 2.99 (t, J = 6.8 Hz, 2H, -CH_2- N_3).

NMR-spectra of synthesized compounds

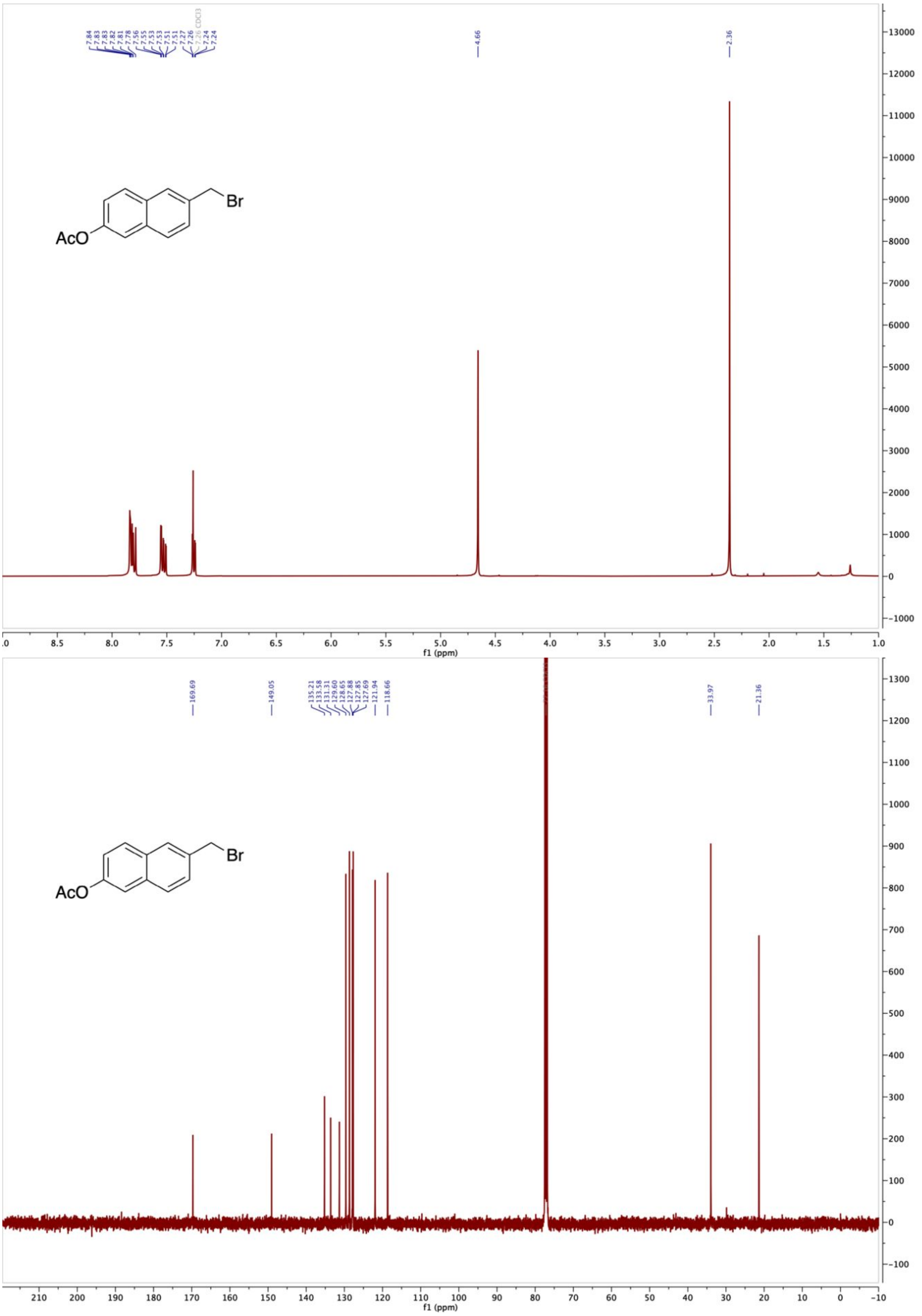

Figure S1 – ^{1}H and ^{13}C spectra for compound **2**

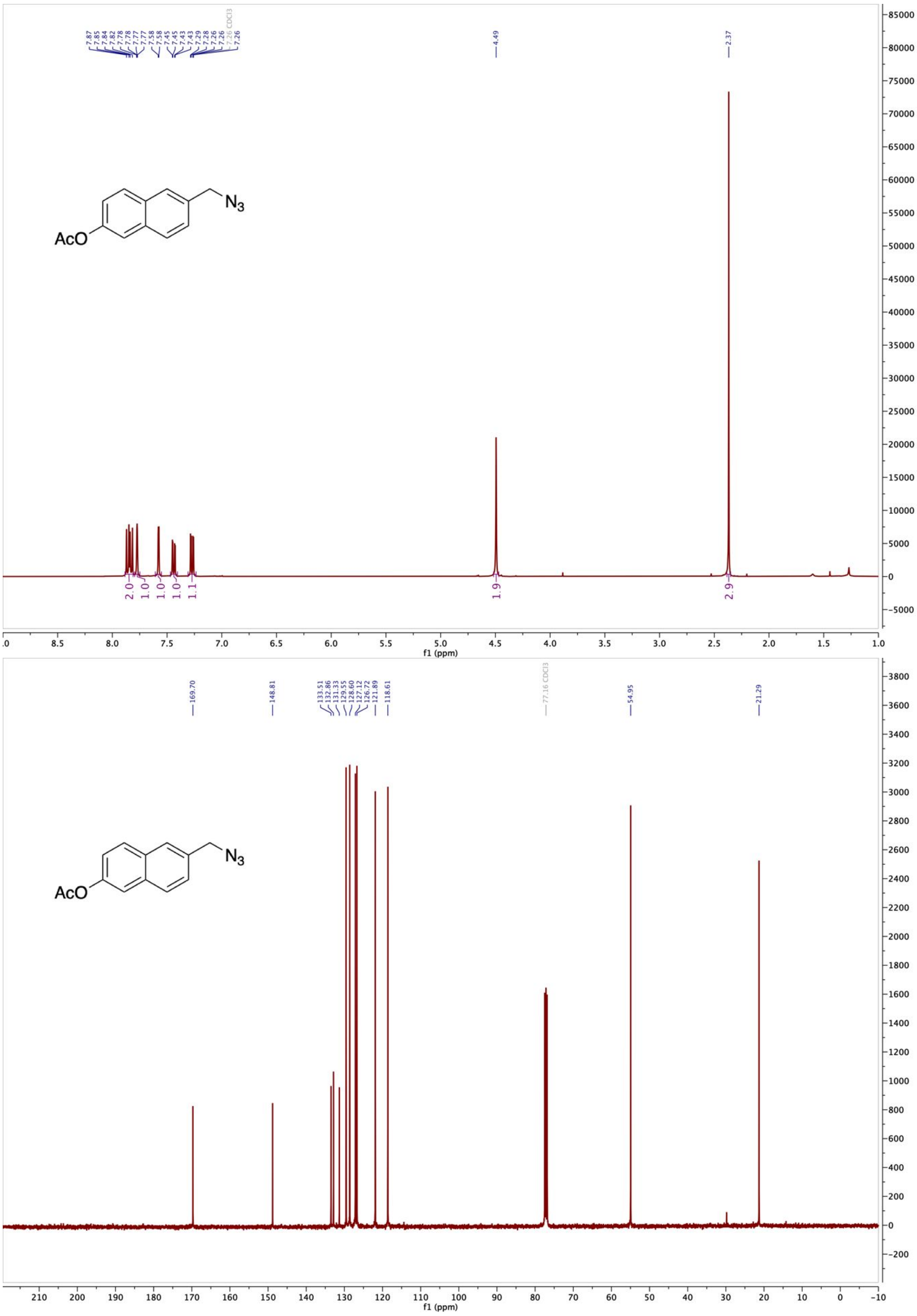

Figure S2 – ^{1}H and ^{13}C spectra for compound **3**

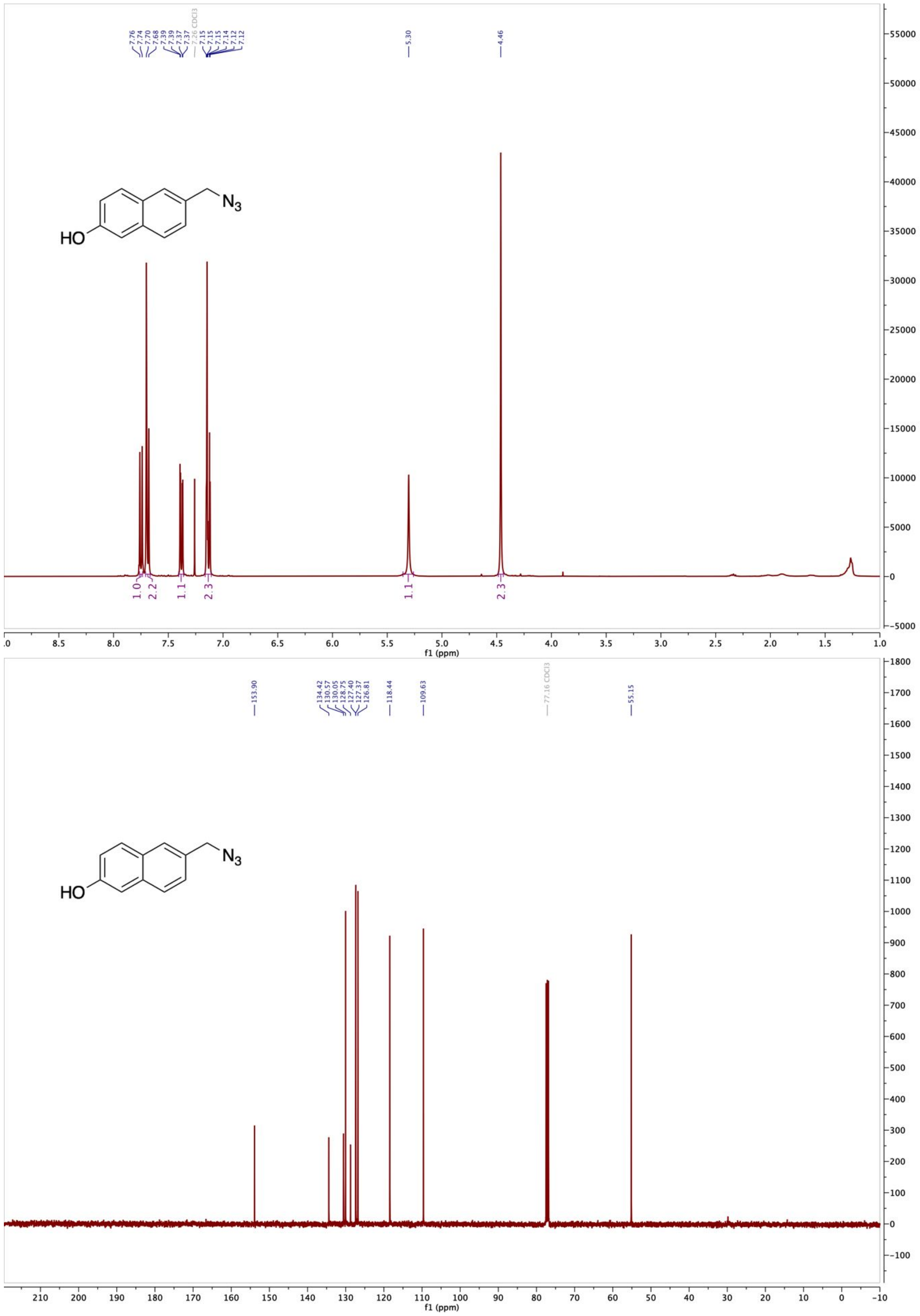

Figure S3 – ^{1}H and ^{13}C spectra for compound **4**

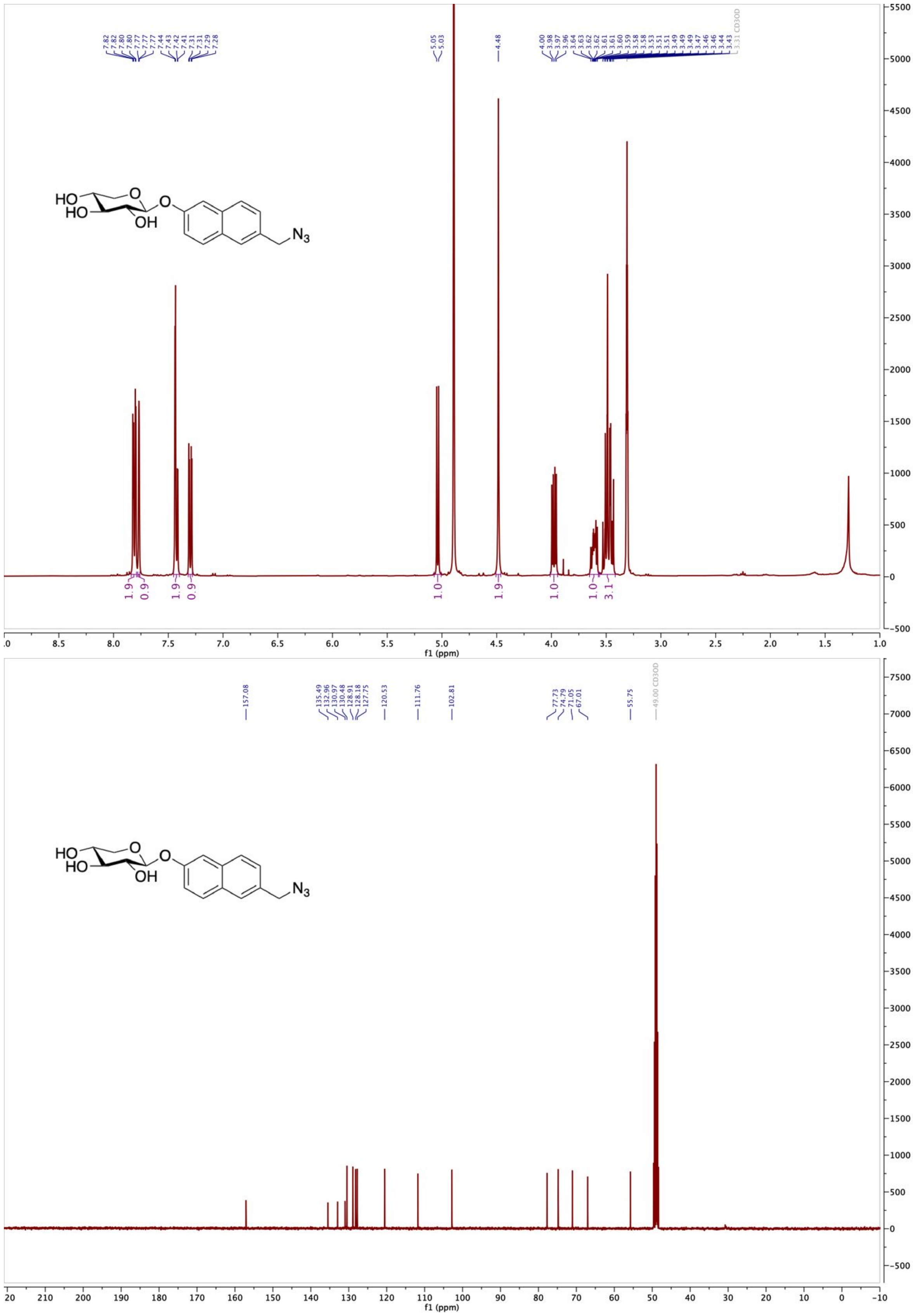

Figure S4 – ^{1}H and ^{13}C spectra for compound **5**

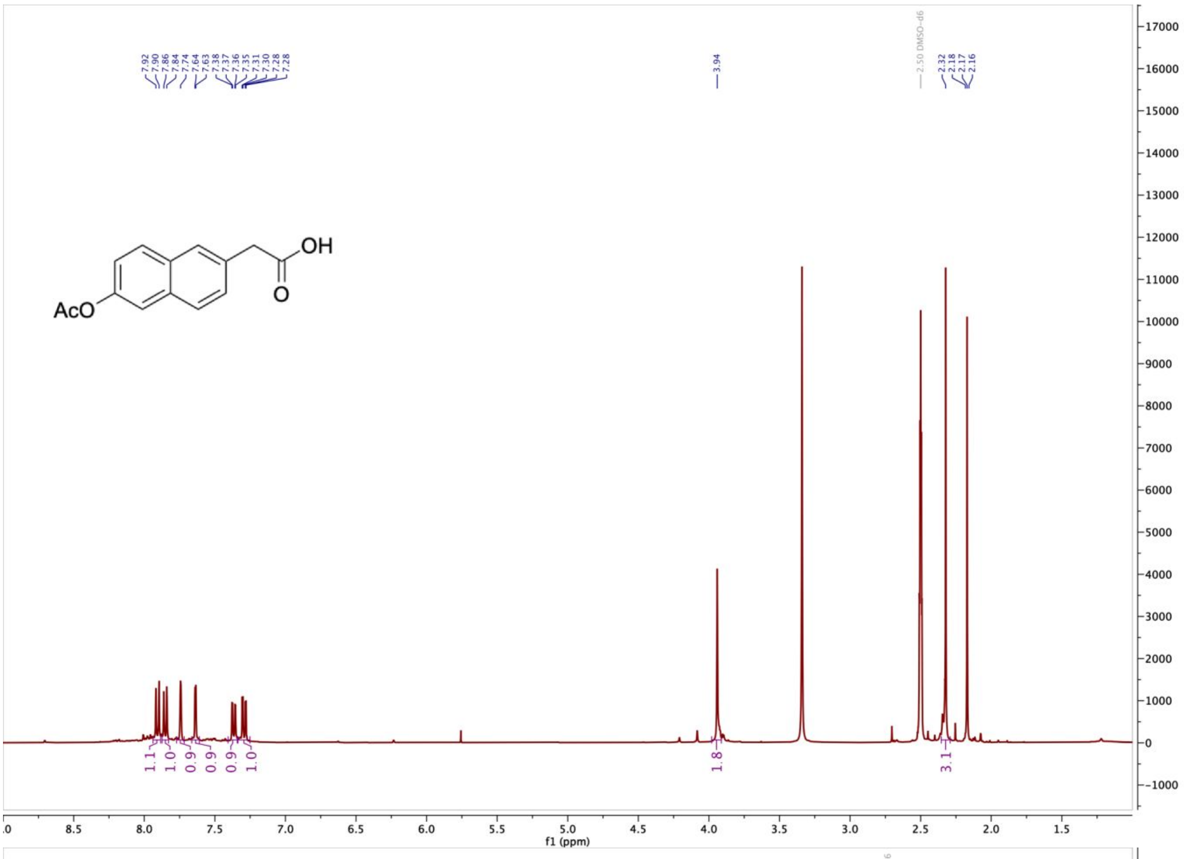

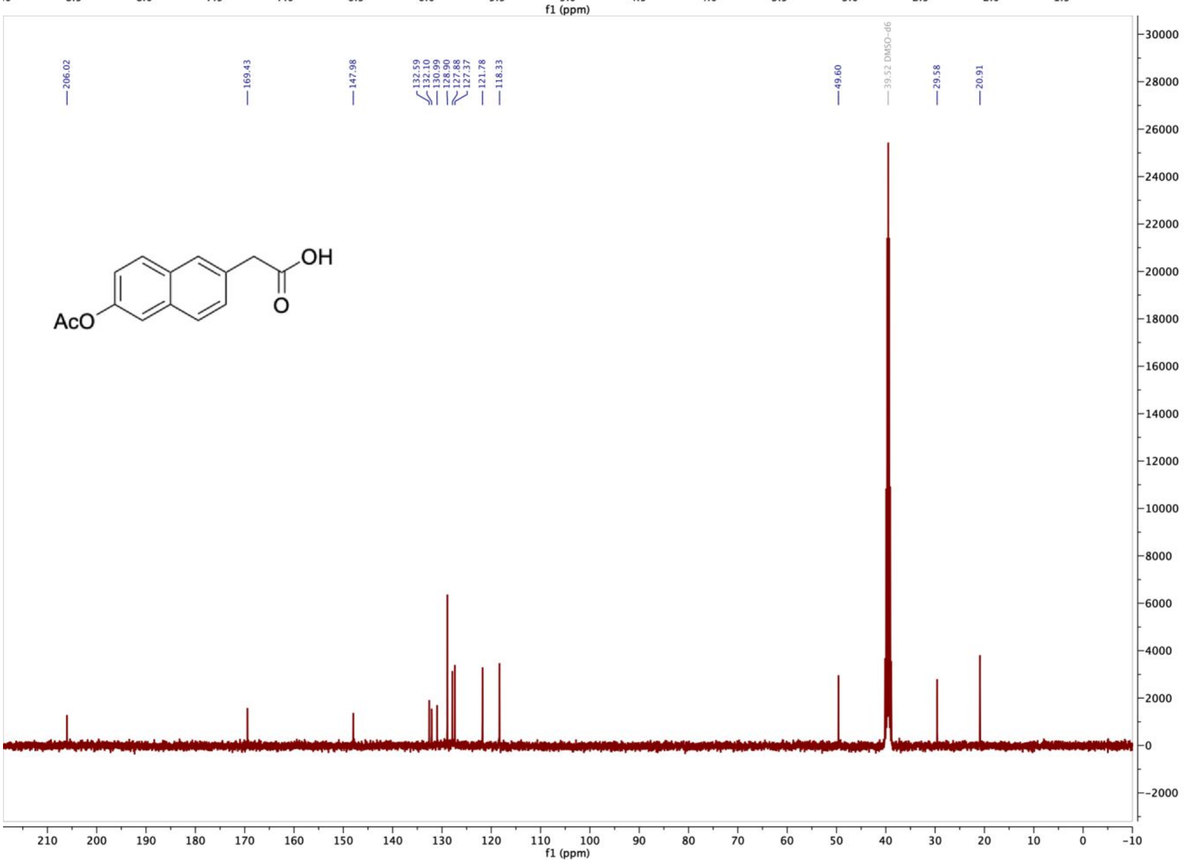

Figure S5 – ^{1}H and ^{13}C spectra for compound **7**

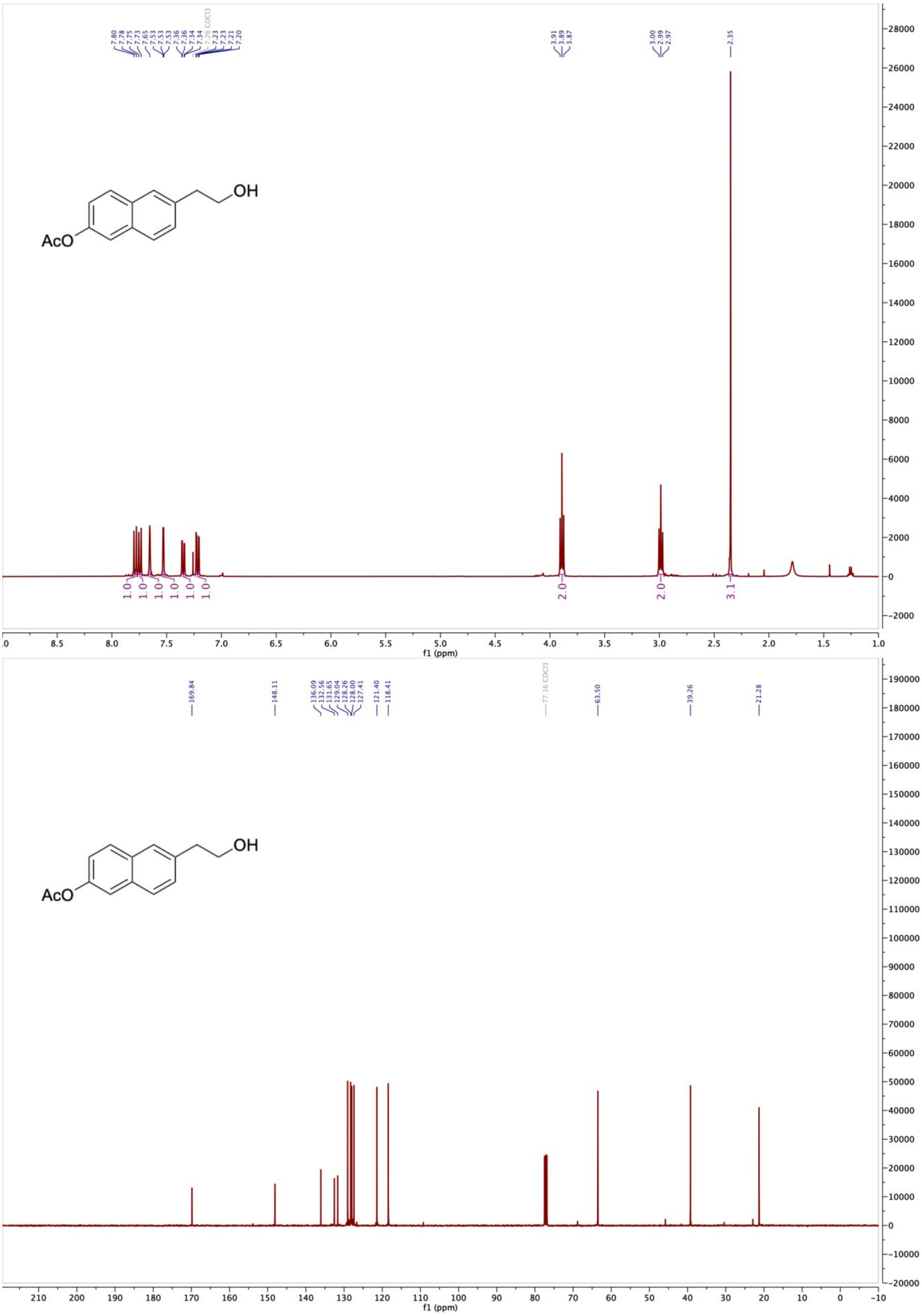

Figure S6 – ^{1}H and ^{13}C spectra for compound **8**

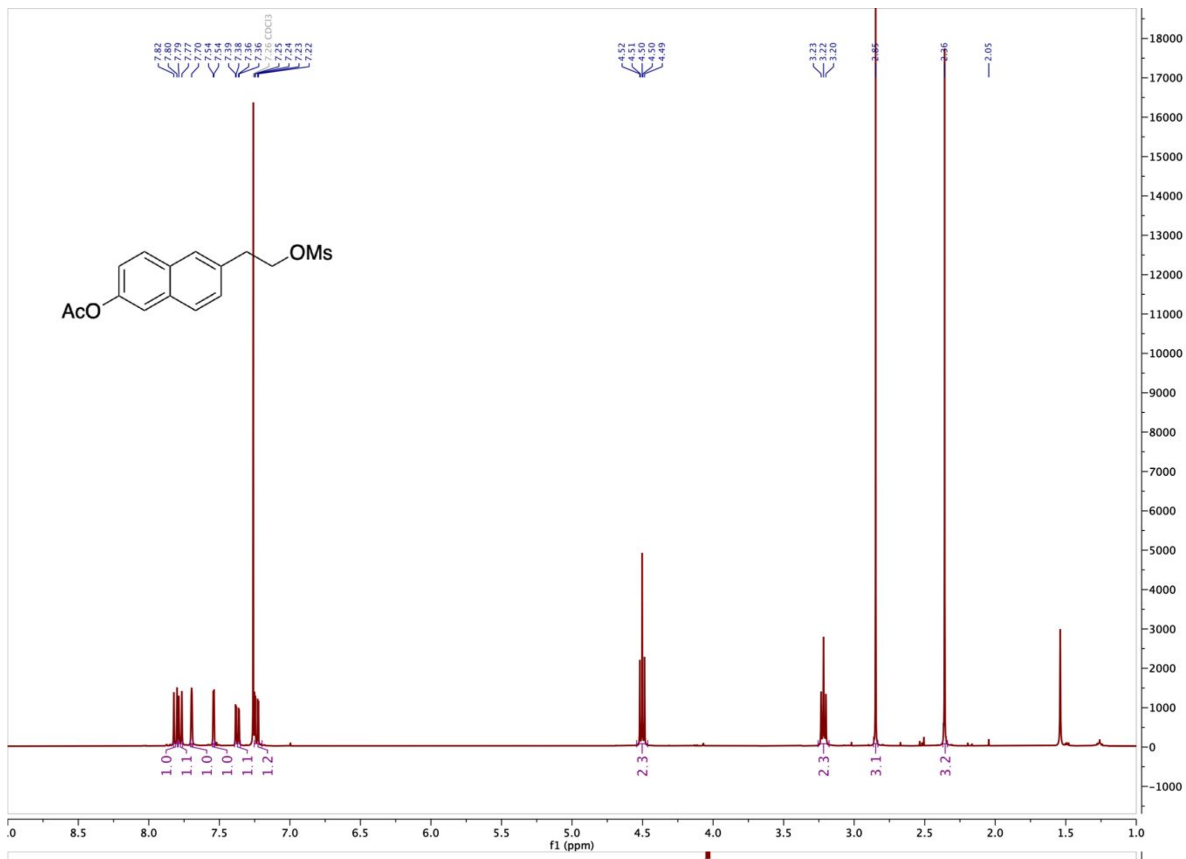

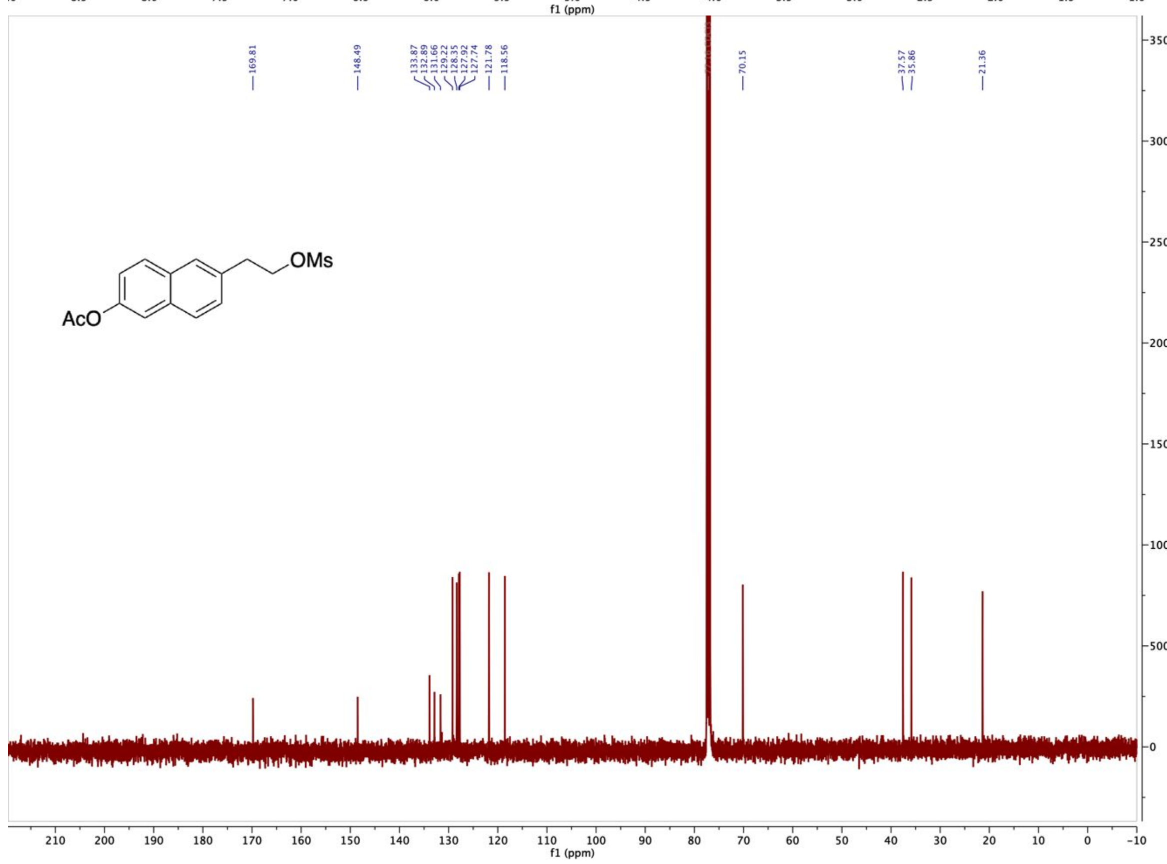

Figure S7 – ^{1}H and ^{13}C spectra for compound **9**

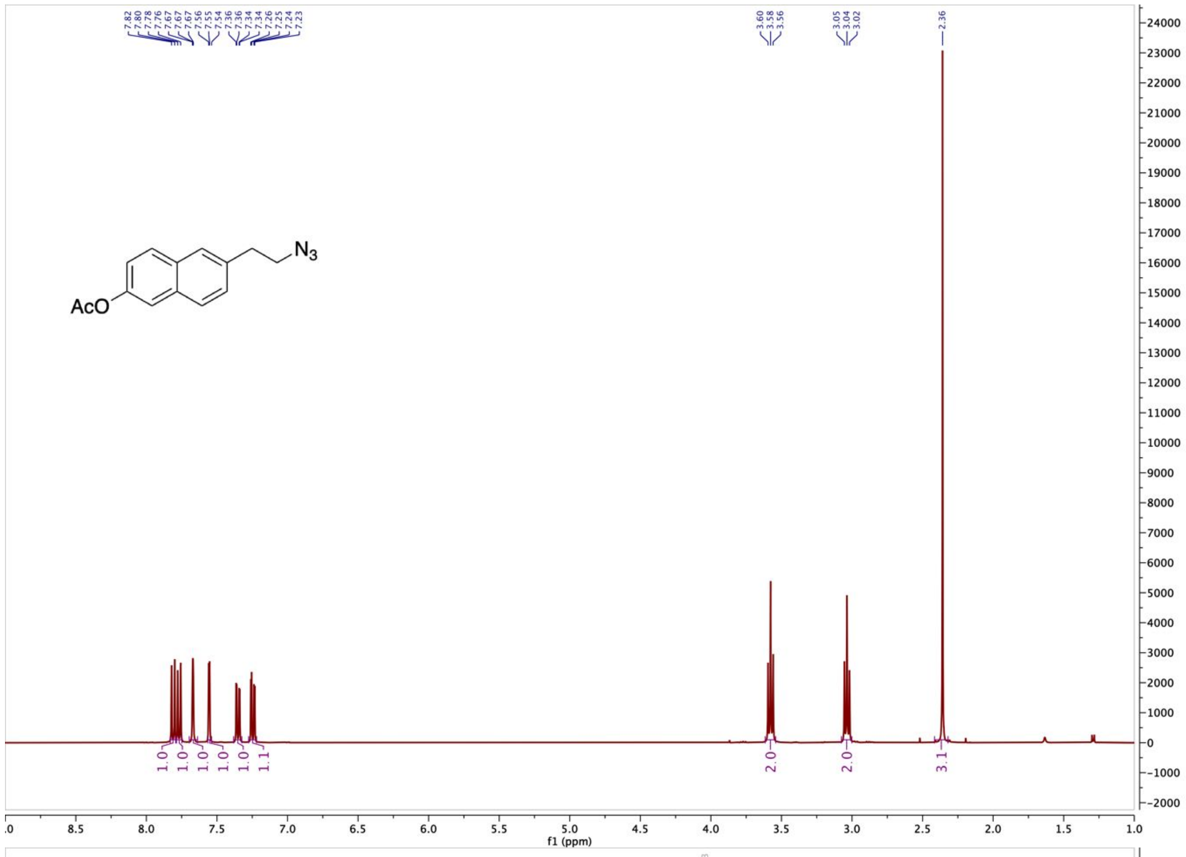

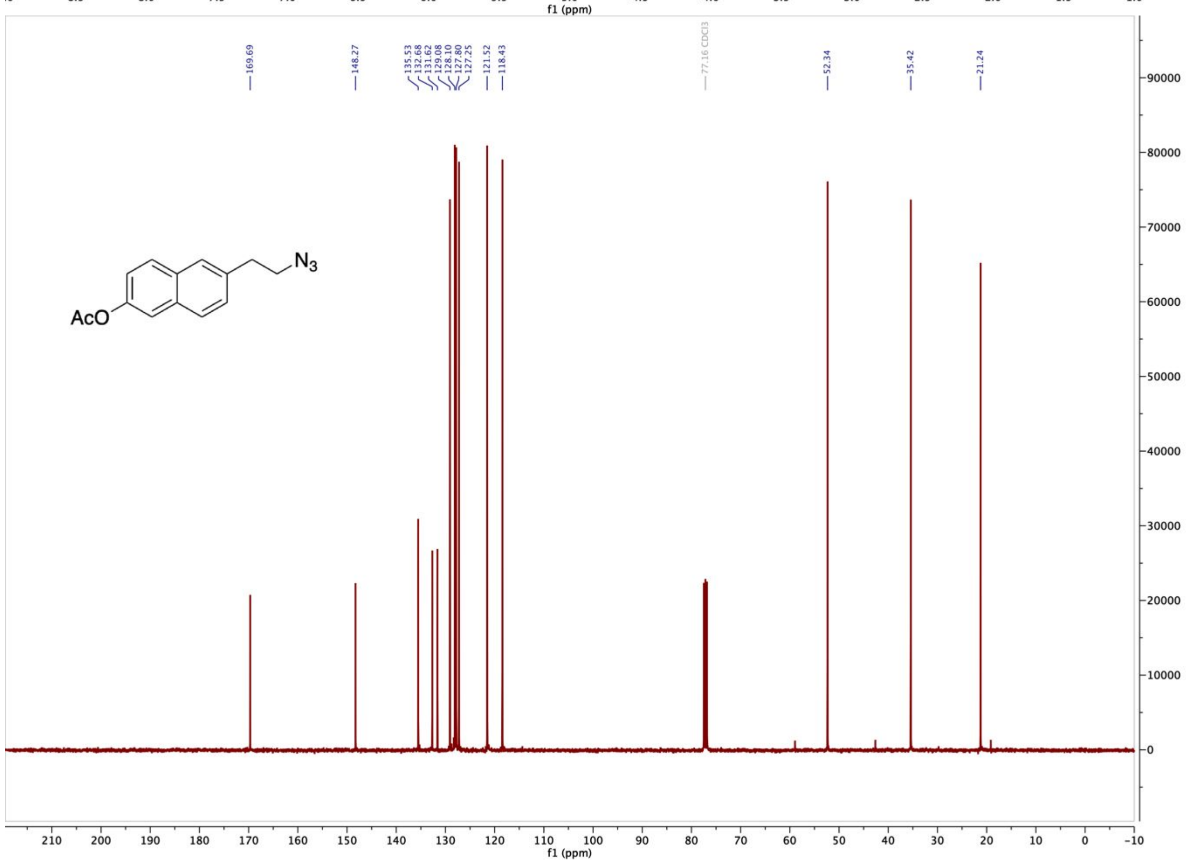

Figure S8 – ^{1}H and ^{13}C spectra for compound **10**

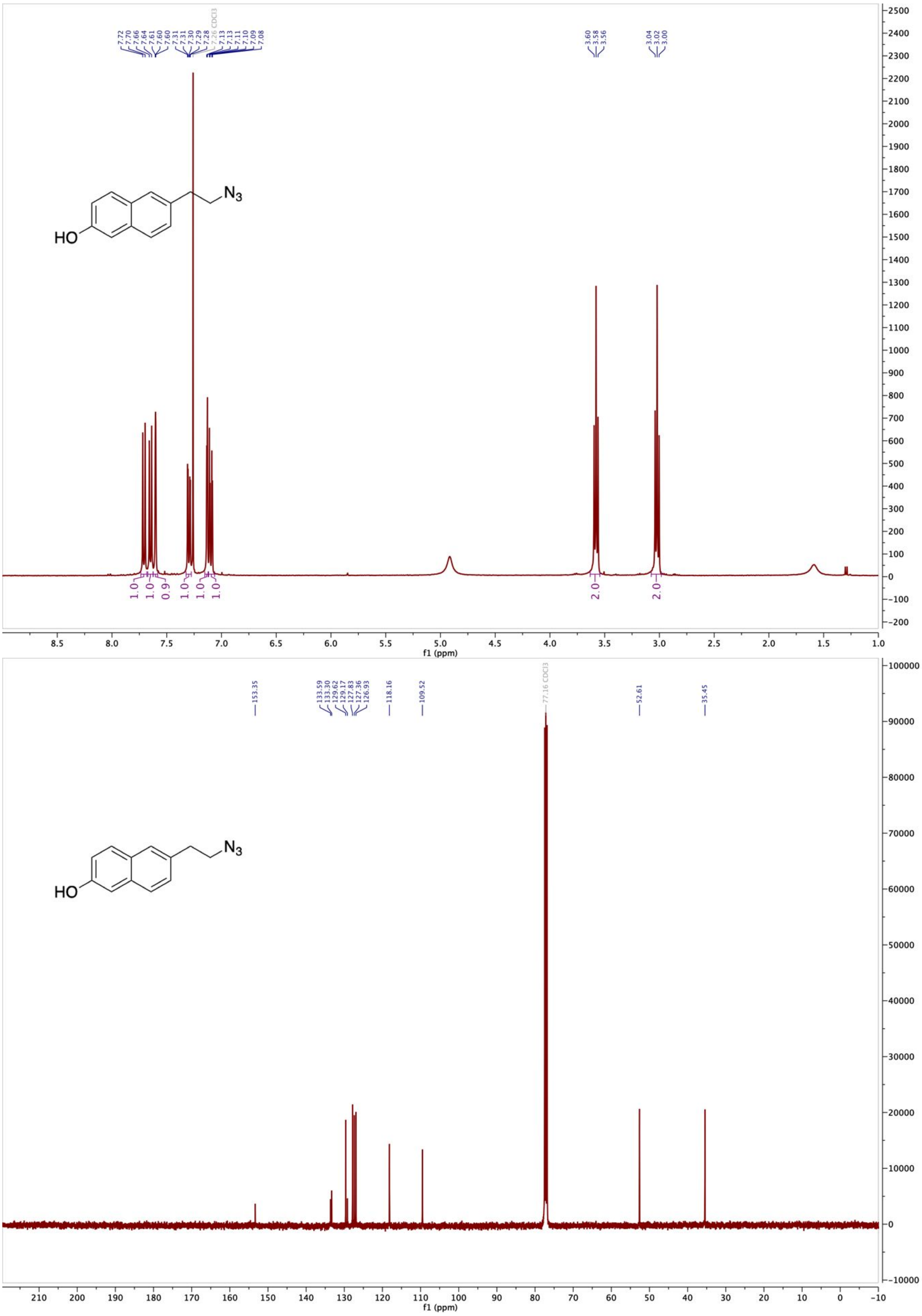

Figure S9 – ^{1}H and ^{13}C spectra for compound **11**

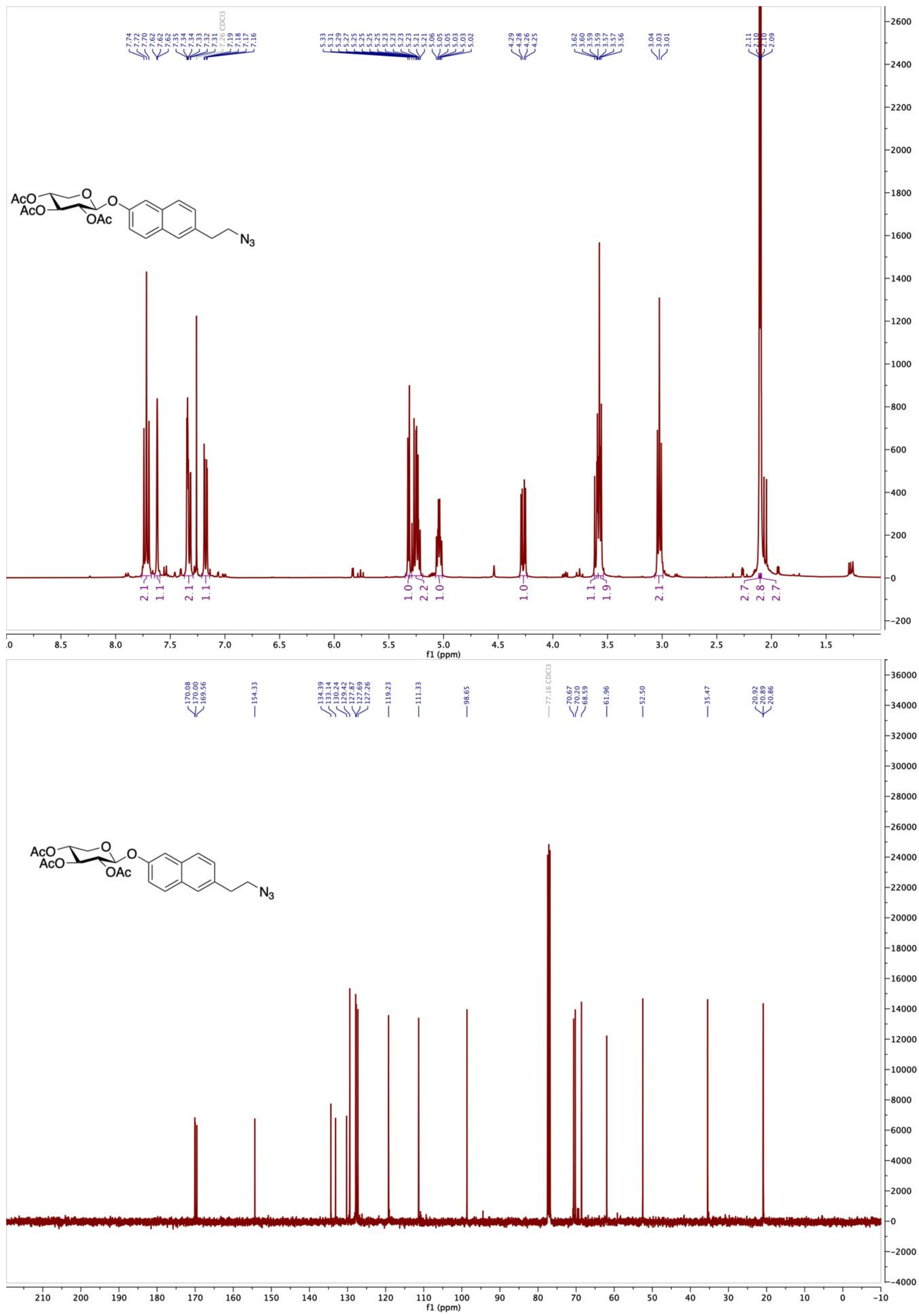

Figure S10 – ^{1}H and ^{13}C spectra for compound **19**

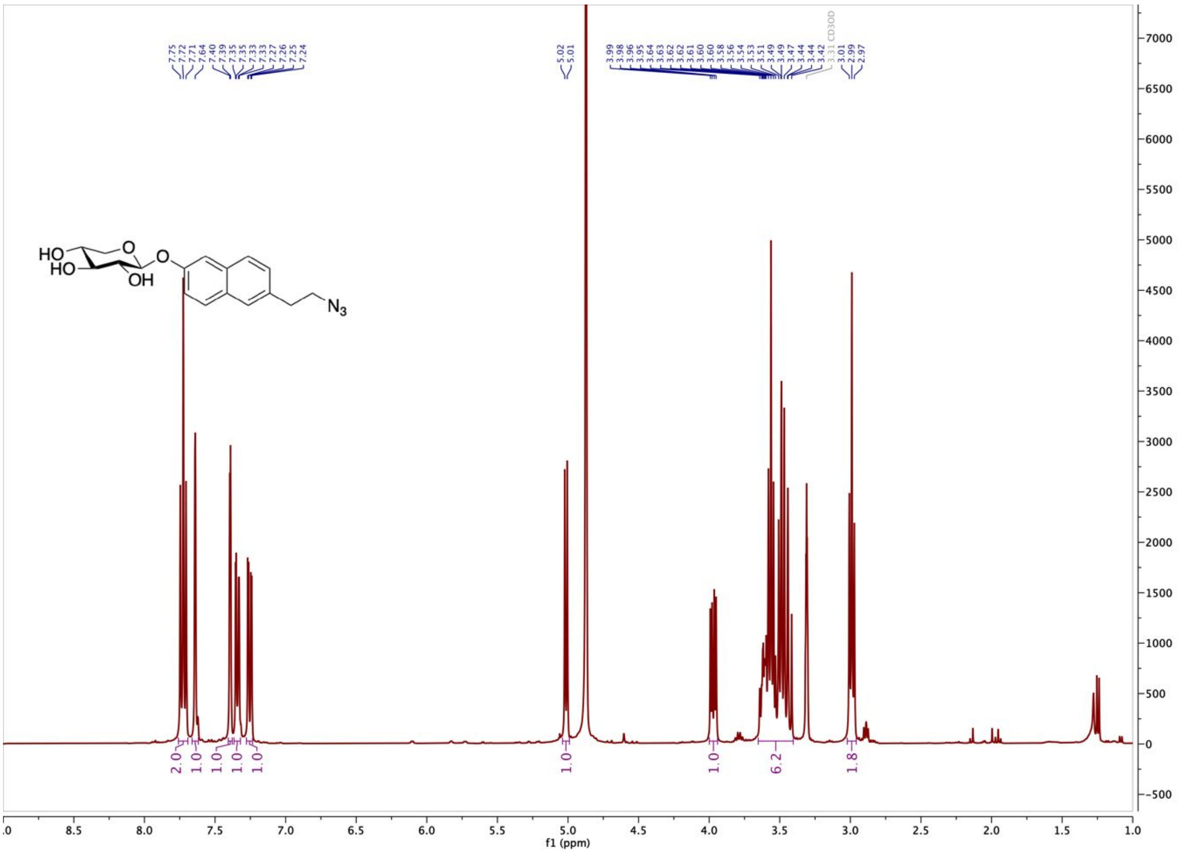

Figure S11 – ^{1}H spectra for compound **12**

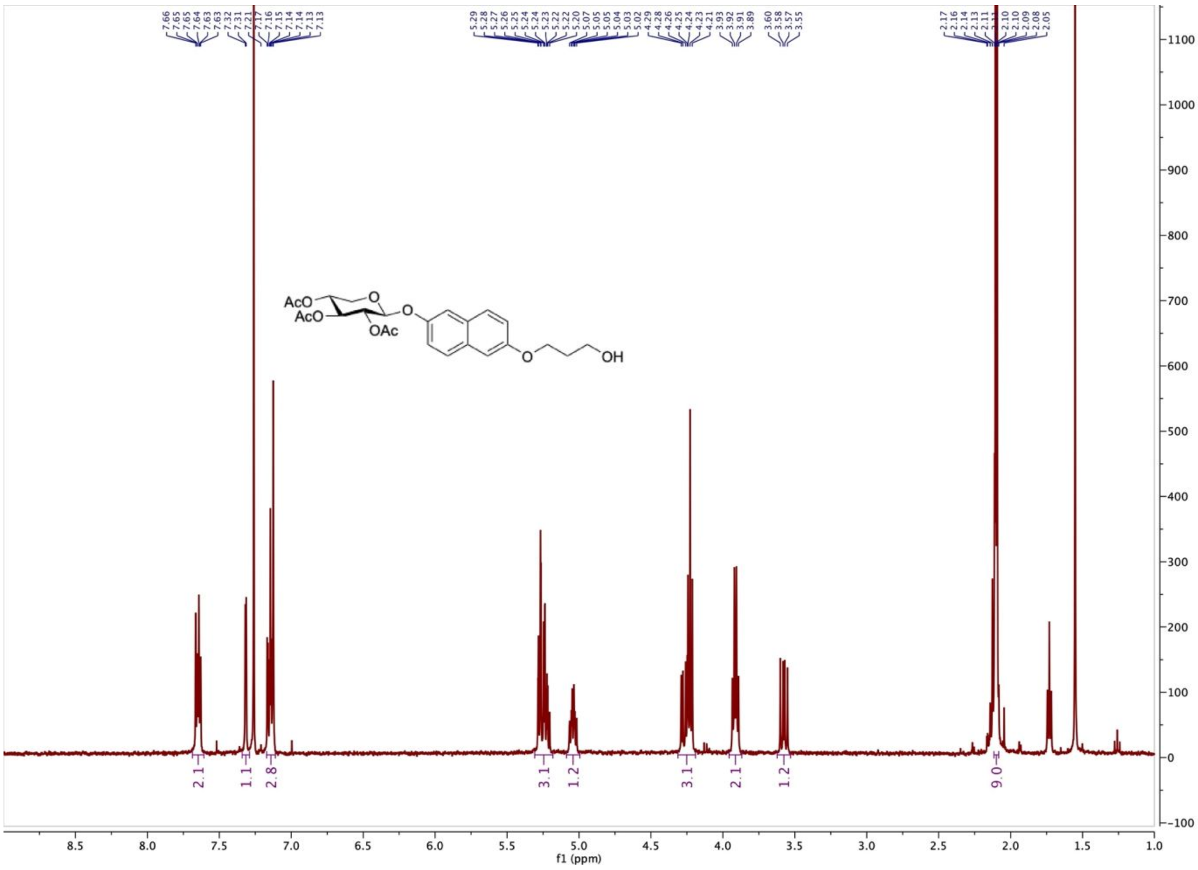

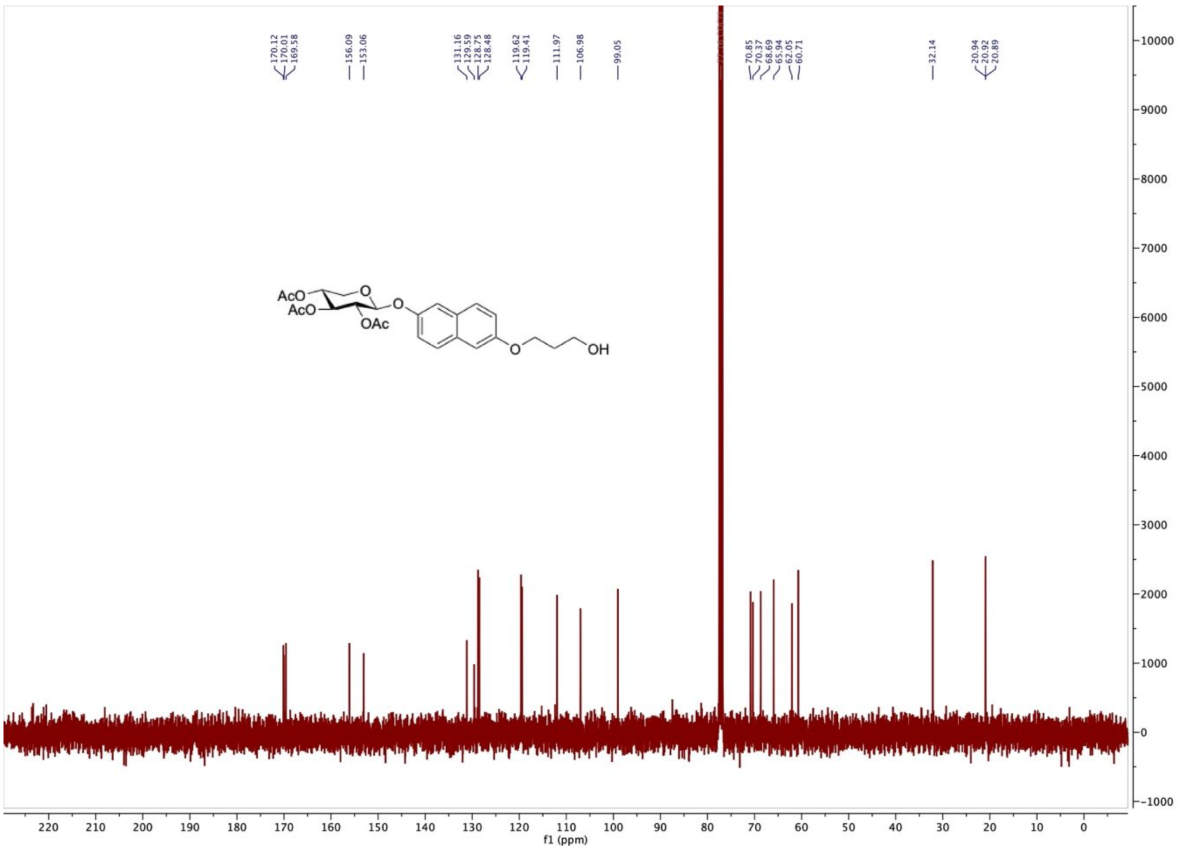

Figure S12 – ^{1}H and ^{13}C spectra for compound **16**

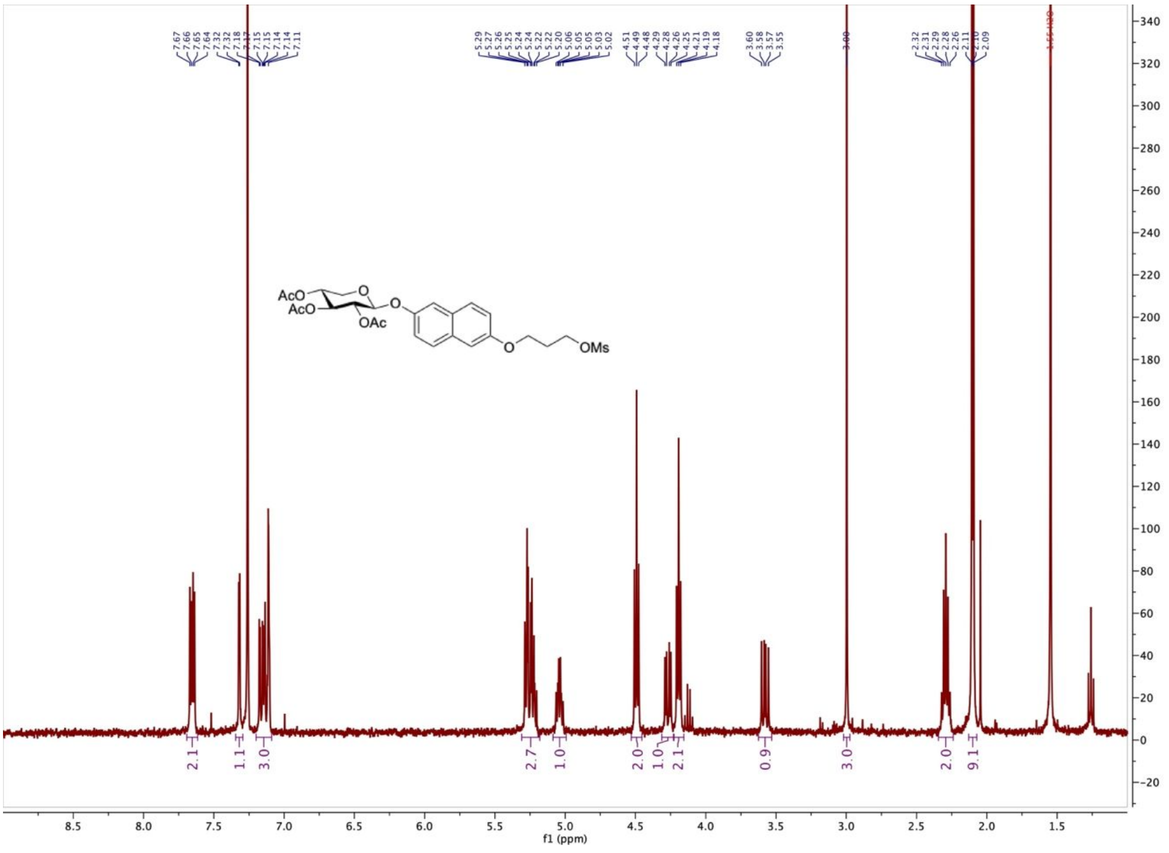

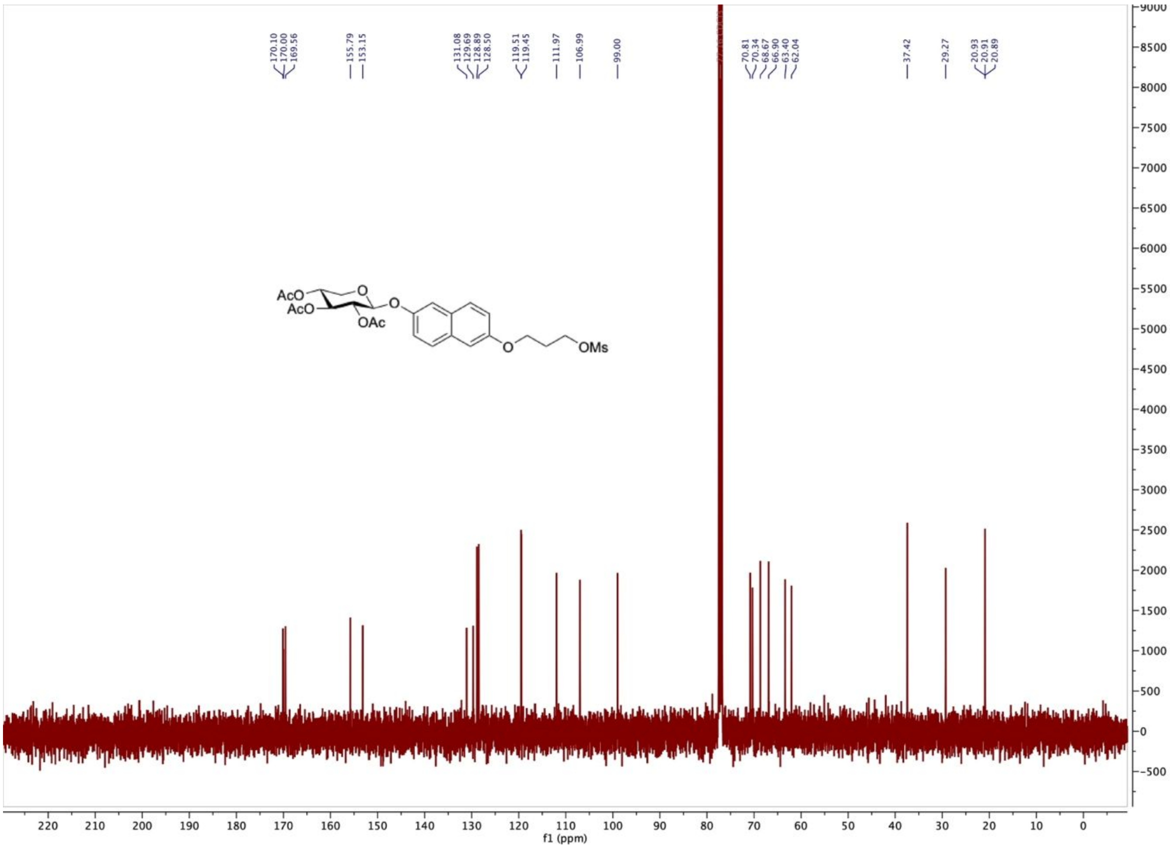

Figure S13 – ^{1}H and ^{13}C spectra for compound **17**

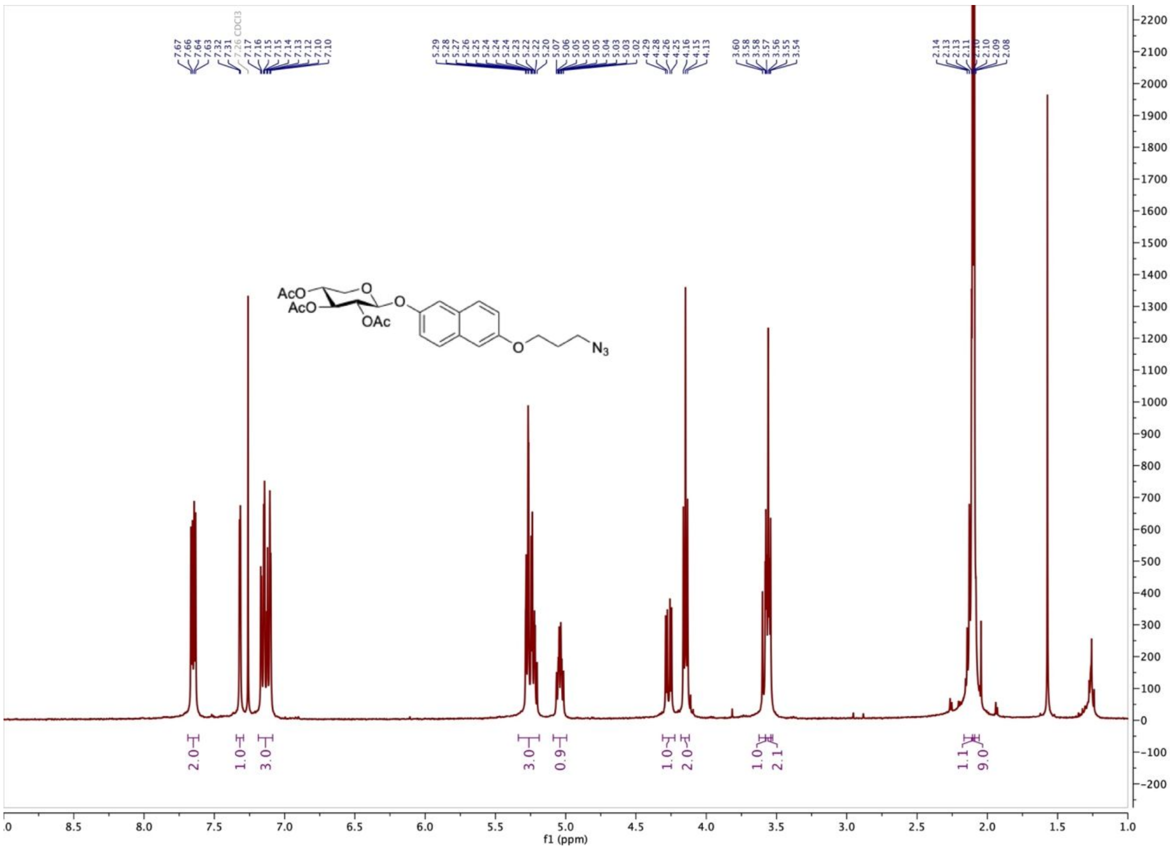

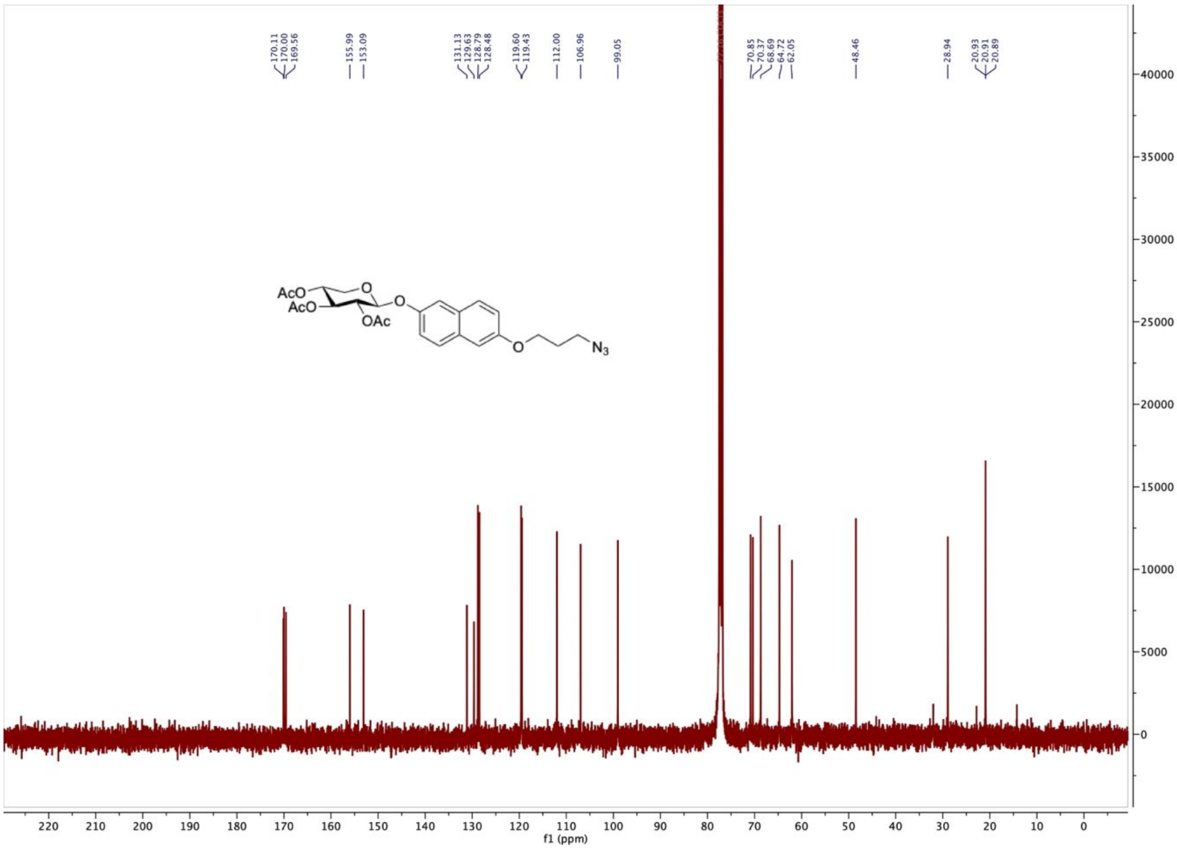

Figure S14 – ^{1}H and ^{13}C spectra for compound **18**

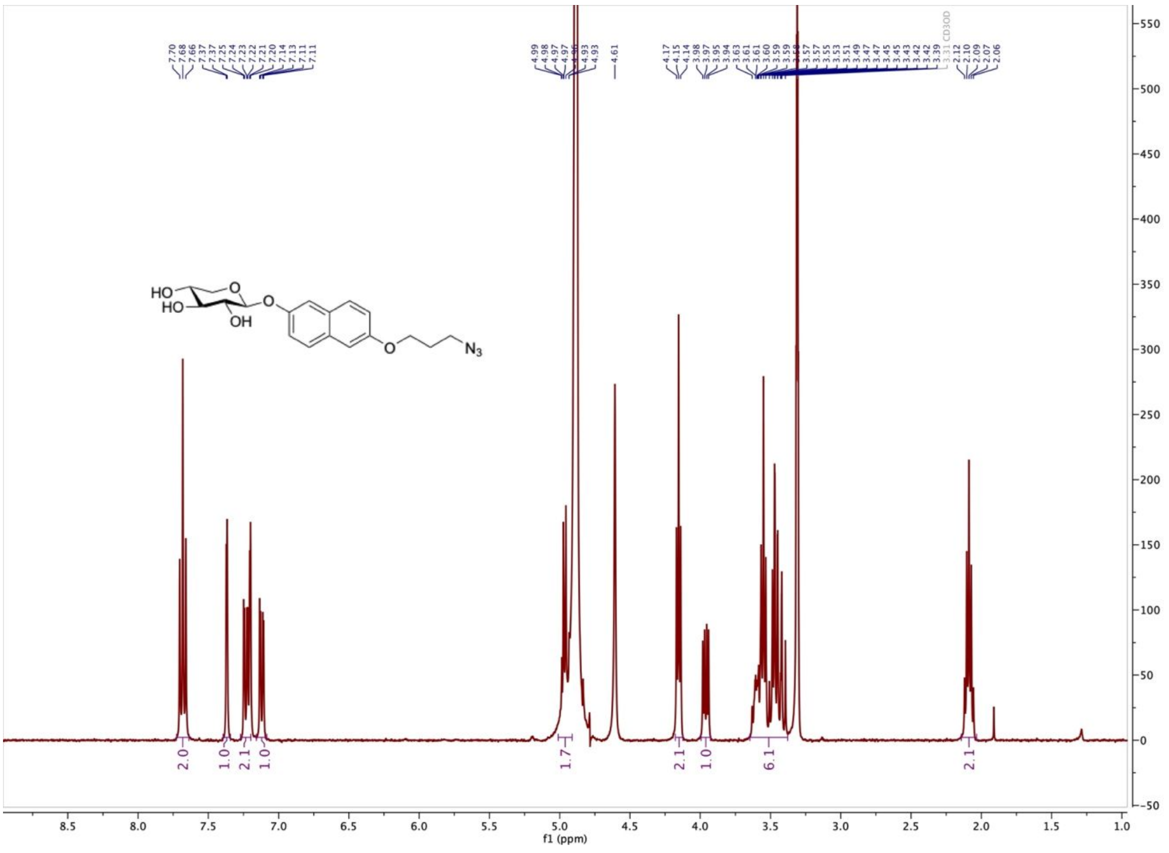

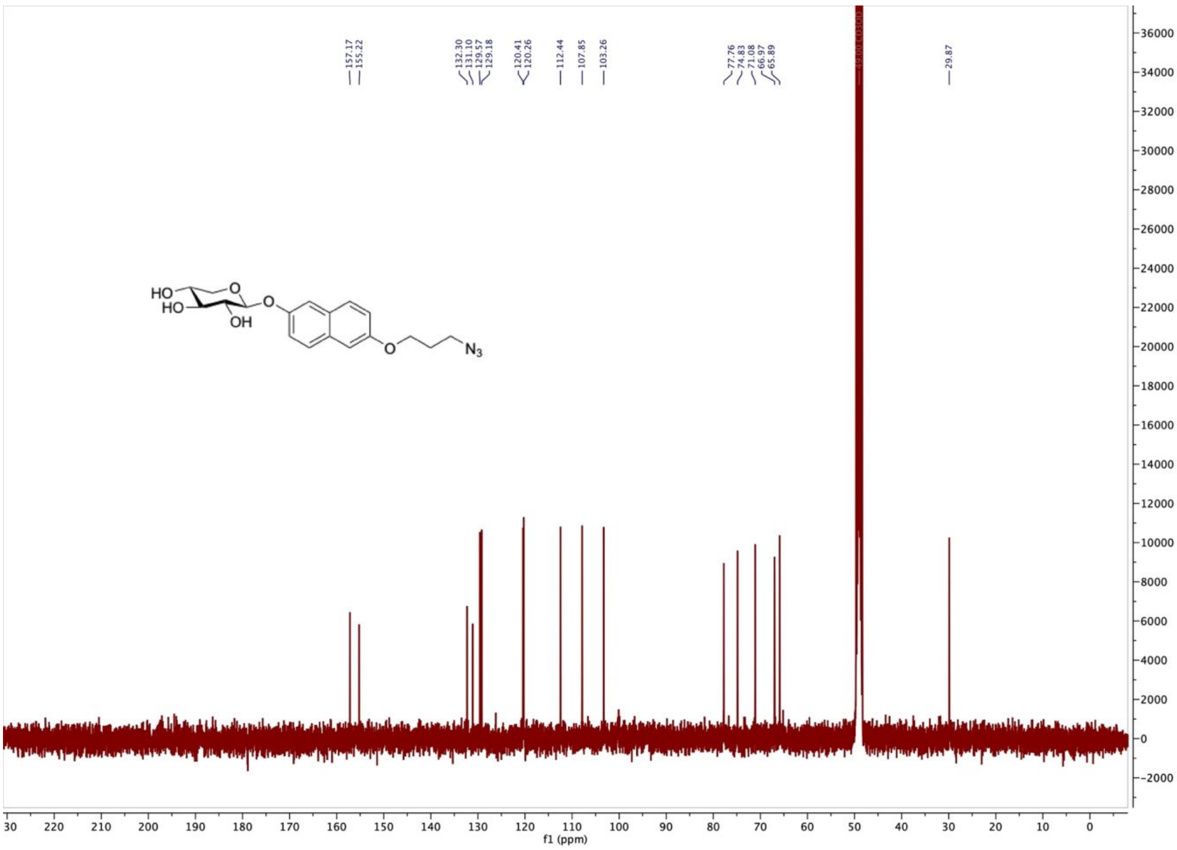

Figure S15 – ^{1}H and ^{13}C spectra for compound **XylNapN3**

References

(1) Jin, Y.; Yang, H.; Wang, C. Nickel-Catalyzed Asymmetric Reductive Arylbenzylation of Unactivated Alkenes. *Org. Lett.* **2020**, *22* (7), 2724–2729.

(2) Johnsson, R.; Mani, K.; Ellervik, U. Evaluation of Fluorescently Labeled Xylopyranosides as Probes for Proteoglycan Biosynthesis. *Bioorg. Med. Chem. Lett.* **2007**, *17* (8), 2338–2341.

(3) Trapella, C.; Fischetti, C.; Pela', M.; Lazzari, I.; Guerrini, R.; Calo', G.; Rizzi, A.; Camarda, V.; Lambert, D. G.; McDonald, J.; Regoli, D.; Salvadori, S. Structure–Activity Studies on the Nociceptin/Orphanin FQ Receptor Antagonist 1-Benzyl-N-{3-[Spiroisobenzofuran-1(3H),4′-Piperidin-1-Yl]Propyl} Pyrrolidine-2-Carboxamide. *Bioorg. Med. Chem.* **2009**, *17* (14), 5080–5095.

(4) Stachtea, X. N.; Tykesson, E.; van Kuppevelt, T. H.; Feinstein, R.; Malmström, A.; Reijmers, R. M.; Maccarana, M. Dermatan Sulfate-Free Mice Display Embryological Defects and Are Neonatal Lethal Despite Normal Lymphoid and Non-Lymphoid Organogenesis. *PLoS One* **2015**, *10* (10), e0140279.
